# *Mycobacterium tuberculosis* suppresses protective Th17 responses during infection

**DOI:** 10.7554/eLife.110008

**Published:** 2026-10-23

**Authors:** Alex Zilinskas, Amir Balakhmet, Douglas Fox, Heyuan Michael Ni, Carolina Agudelo, Helia Samani, Sarah A Stanley

**Affiliations:** 1 Division of Immunology and Molecular Medicine, Department of Molecular and Cell Biology, University of California, Berkeley Berkeley United States; 2 Division of Infectious Disease and Vaccinology, School of Public Health, University of California, Berkeley Berkeley United States; University of the Witwatersrand South Africa; University of the Witwatersrand South Africa

**Keywords:** *Mycobacterium tuberculosis*, Th17, ESX-1, Mouse

## Abstract

*Mycobacterium tuberculosis* (Mtb) causes more deaths annually than any other pathogen, yet an effective vaccine remains elusive. IFN-γ-producing Th1 CD4+ T cells are necessary but insufficient for protection against infection. In humans, the development of IL-17A-producing Th17 T cells correlates with protection; however, not all individuals develop a Th17 response. In mice, experimental vaccines can elicit protective Th17 cells, yet Th17s are rare in primary infection. Why Mtb fails to consistently elicit Th17s is unknown. Here, we identify factors suppressing Th17 responses during primary infection. We demonstrate that the lack of Th17 induction is independent of route and duration. Next, using *Tbx21*-deficient mice, we show that Mtb drives a Th1 response that is only partially protective and limits Th17 cell production in an IFN-γ-independent manner. We further reveal that the ESX-1 type VII secretion system and lipid phthiocerol dimycocerosate (PDIM) suppress Th17 responses. Infection with ESX-1 or PDIM mutants results in significantly increased Th17 T cells and IL-17A cytokine in the lungs, and infection of IL-17A-deficient animals partially restores virulence of ESX-1 and PDIM mutants. Although the ESX-1 secretion system and lipid PDIM elicit type I IFN, which can suppress Th17 differentiation, we find that suppression of Th17 is independent of type I IFN. Instead, ESX-1 and PDIM suppress production of IL-23, a cytokine that promotes Th17 differentiation, in dendritic cells found in mediastinal lymph nodes during Mtb infection. These findings define a new function of the ESX-1 secretion system and PDIM in Mtb virulence, a long-standing question in tuberculosis research.

## Introduction

*Mycobacterium tuberculosis* (Mtb), the causative agent of tuberculosis (TB), remains one of the leading infectious disease killers worldwide, with 10.8 million new cases and 1.25 million deaths reported in 2021 (World Health Organization, 2022). The current vaccine, *Mycobacterium bovis bacillus Calmette–Guérin* (BCG), protects against childhood TB, but has highly variable efficacy, wanes over time, and provides minimal protection against adult pulmonary TB (World Health Organization, 2022; Fine, 1995; Kumar, 2022). Although there are 17 vaccine candidates currently in clinical trials, achieving robust vaccine-elicited immunity remains a major challenge (Zhou and Zhang, 2023).

Interferon-γ (IFN-γ) produced by type 1 T helper (Th1) cells is essential for control of Mtb infection both in animal models and in humans (Flynn et al., 1993; Casanova and Abel, 2002). However, the MVA85A vaccine, despite inducing robust Th1 responses as intended, failed to provide protection against Mtb infection (Tameris et al., 2013). Patients with active TB can have high levels of IFN-γ in the lungs despite being unable to control infection (Zhang et al., 2021). Thus, Th1 cells are necessary but not sufficient for controlling Mtb infection, suggesting that other factors are important for effective immunity. Without a comprehensive understanding of which types and specific subsets of T helper cells are essential to limit Mtb infection, developing an effective Mtb vaccine with consistent high efficacy will remain elusive.

Type 17 T helper (Th17) cells are known for their association with mucosal sites, including the lungs, where they have been shown to provide protection against several bacterial and fungal respiratory pathogens (Guglani and Khader, 2010). This protection relies on IL-17A production by Th17 cells (Gaffen, 2009). Many factors determine whether Th17 cells are elicited by infection. Antigen-presenting cells drive the polarization of naïve T cells into Th17 cells by producing cytokines, including IL-1β, IL-6, TGF-β, and IL-23. Activation of the RORγT transcription factor is essential for Th17 polarization (Lazarevic et al., 2011). There is also evidence that the route of infection influences whether a Th17 response is elicited as infection with *Listeria monocytogenes* via the intranasal, but not intravenous route is required to observe a Th17 response to infection (Pepper et al., 2010).

Th17 differentiation is opposed by Th1 differentiation via multiple mechanisms. Tbet, the transcription factor essential for commitment to the Th1 lineage, has been shown to prevent Runx1 activation, thereby preventing Runx1-mediated transactivation of RORγT and Th17 differentiation (Lazarevic et al., 2011). Additionally, IFN-γ, the signature cytokine of Th1s, inhibits Th17 differentiation and function via activation of STAT1, independent of Tbet (Yeh et al., 2014). IFN-γ inhibits Th17 differentiation and function via Tbet-dependent and Tbet-independent mechanisms (Yeh et al., 2014). Finally, production of type I IFN also can suppress Th17 differentiation (Guo et al., 2008). Thus, several hallmarks of Mtb infection, namely strong Th1 responses, IFN-γ production, and type I interferon production, could limit differentiation of Th17s.

Increasing evidence that Th17 cells correlate with protection against Mtb infection both in the non-human primate model (Gideon et al., 2022) and in humans (Ogongo et al., 2024; Sun et al., 2024; Nathan et al., 2021) suggests that Th17 cells are an important component of protective immunity to Mtb. In mice, Th17 cells are rarely observed during primary Mtb infection, and the conditions under which Th17 cells are induced during infection remain unclear (Gopal et al., 2014). However, experimental TB vaccines, particularly when administered via a mucosal route, elicit Th17 cells that can confer protection against subsequent challenge in mice (Khader et al., 2007; Aguilo et al., 2016; Jong et al., 2022), demonstrating that Th17 cells can be protective against Mtb infection. Why Mtb infection does not robustly elicit Th17 cells during primary infection, despite causing infection at mucosal surfaces via airway infection, is unclear.

The ESX-1 type VII secretion system is a fundamental determinant of Mtb virulence (Stanley et al., 2003). The ESX-1 secretion system has been demonstrated to subvert and/or manipulate immune responses, including by perforating phagosomal membranes, inducing detrimental host type I interferon, and inducing autophagy (Stanley et al., 2007; Watson et al., 2015; Watson et al., 2012). As much of the work examining ESX-1 function has been conducted in vitro, we lack a clear understanding of all the factors that attenuate ESX-1 mutants during in vivo infection. Interestingly, the Mtb lipid phthiocerol dimycocerosate (PDIM) is also required for virulence and has been linked to ESX-1 function through induction of type I IFN, which can suppress Th17 responses (Cox et al., 1999; Barczak et al., 2017). Furthermore, the ESX-1 secretion system was shown to suppress production of IL-12 p40 (Stanley et al., 2003), a subunit shared with IL-23, which promotes Th17 differentiation. These data suggest that Mtb virulence factors, including ESX-1 and PDIM, may suppress the emergence of a protective Th17 response during Mtb infection.

Here we show that the induction of Th17 cells during Mtb infection is suppressed by multiple mechanisms. First, we show that the absence of a Th17 response during primary infection is not dependent on duration or route of infection. Instead, we show that mice lacking *Tbx21* (*Tbx21*^–/–^) produce Th17 cells after Mtb infection, and that these cells provide IL-17A-dependent protection against early Mtb infection. This suppression of Th17 production by Th1 cells is independent of IFN-γ. Furthermore, we observe a strong Th17 response during infection with ΔESX-1 or PDIM-lacking Mtb in mice, suggesting that these virulence factors function to suppress Th17 differentiation. Surprisingly, the ability of ESX-1/PDIM to suppress Th17 differentiation is not related to their enhanced induction of type I IFN. Instead, we show that during infection with ΔESX-1 or PDIM-lacking Mtb strains, type 2 conventional dendritic cells (CD11b^+^ DCs, cDC2s), which are known to support Th17 differentiation, produce higher levels of IL-23 secretion in draining lymph nodes of Mtb-infected mice (Schlitzer et al., 2013). These results demonstrate that Mtb utilizes the ESX-1 secretion system and PDIM to bias host T helper response toward Th1s and away from Th17s.

## Results

### Th17 induction during *M. tuberculosis* infection does not depend on duration or route of infection

**Figure 1.**
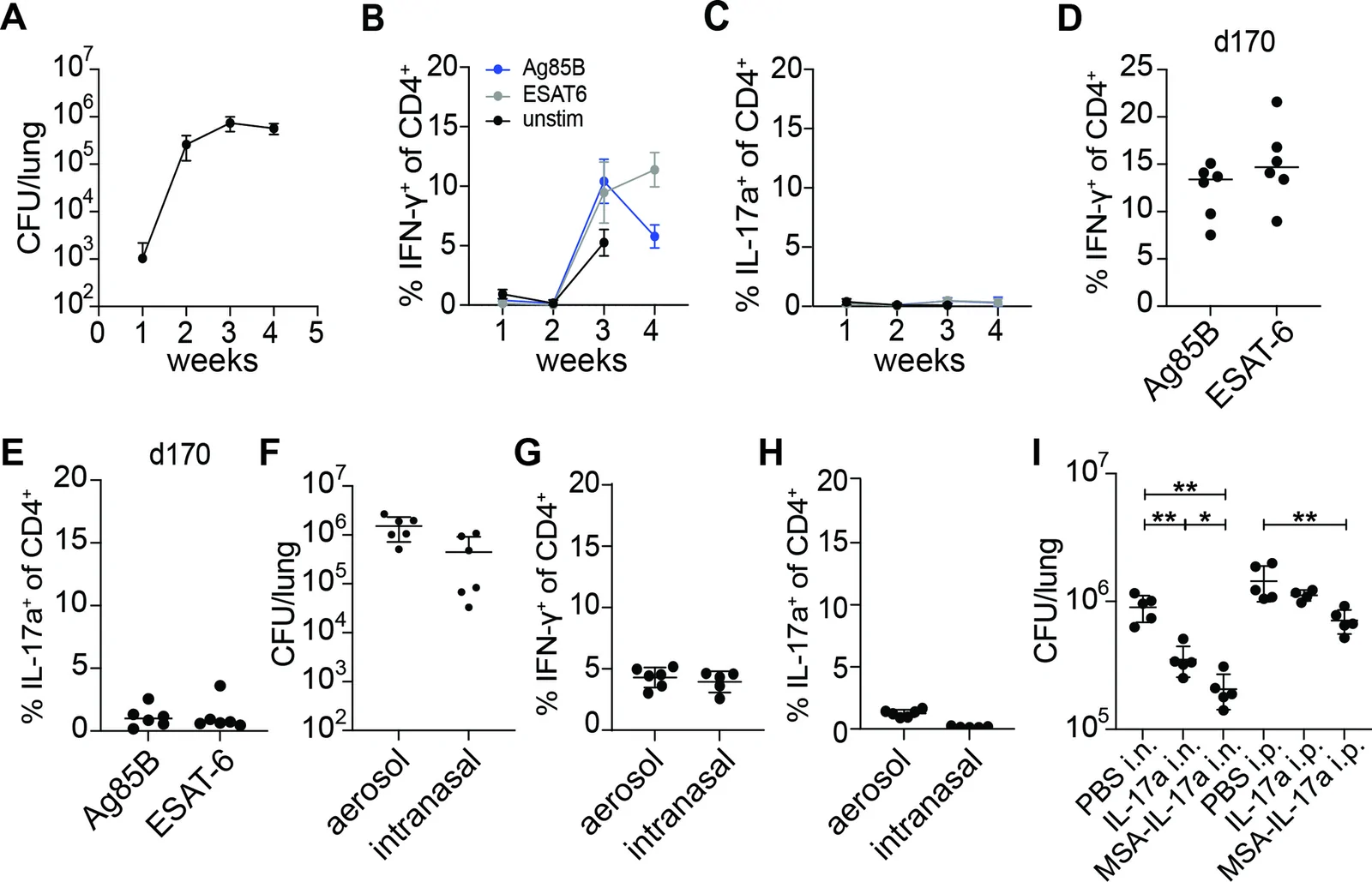
Lack of Th17 induction during WT *M.* *tuberculosis* infection is not due to duration or route of infection. (**A–C**) WT B6 mice were aerosol infected with WT *M. tuberculosis* Erdman strain, and lungs were evaluated at 7, 14, 21, and 28 days post infection (dpi) for (**A**) bacterial burdens by CFU, (**B**) IFN-γ^+^ CD4^+^ T cells, and (**C**) IL-17A^+^ CD4^+^ T cells using flow cytometry. Cytokines were measured using ICS after restimulation with Ag85b or ESAT-6 peptide pools. (**D, E**) Mice were infected and analyzed at 170 dpi for (**D**) IFN-γ^+^ CD4^+^ T cells and (**E**) IL-17A^+^ CD4^+^ T cells in lungs. (**F–H**) WT B6 mice were either aerosol infected or intranasally infected with WT *M. tuberculosis* Erdman strain and evaluated 28 dpi for (**F**) bacterial burdens in lungs by CFU, (**G**) IFN-γ^+^ CD4^+^ T cells, and (**H**) IL-17A^+^ CD4^+^ T cells in lungs by flow cytometry. Cytokines were measured using ICS after restimulation with ESAT-6 peptide pool. (**I**) WT B6 mice were aerosol infected with WT *M. tuberculosis* and treated with PBS control, recombinant murine IL-17A (IL-17A), or recombinant murine IL-17A fused to murine serum albumin (MSA-IL-17A) daily either using the intranasal (i.n.) or intraperitoneal (i.p.) routes between days 11–20 post infection. CFU in lungs was measured at 21 dpi. Results are representative of at least two biological replicates. For all graphs, each dot represents an individual mouse. *p<0.05, **p<0.01 (unpaired nonparametric Mann–Whitney *U* test).

We first tested whether the duration or route of infection has an impact on the generation of Th17 cells during Mtb infection. To test for induction of Th17 cells, we infected wild-type mice with ~100 CFU of WT *M. tuberculosis* via the aerosol route, a standard dose for the mouse model (Soldevilla et al., 2022). As previously published by multiple groups, we do not observe Th17 cells during the first month after Mtb infection of mice (Gopal et al., 2014; Khader et al., 2007; Aguilo et al., 2016; Jong et al., 2022). At 4 weeks post infection, we observed the expected plateau of CFU in the lungs of infected mice indicating the onset of T cell-based control of infection (Figure 1A). With stimulation using peptide pools from model antigens ESAT-6 and Antigen 85B (Ag85B) (Andersen et al., 1995; Bekmurzayeva et al., 2013; Karbalaei Zadeh Babaki et al., 2017), we observed only IFN-γ^+^ CD4^+^ (Th1) T cells (Figure 1B) and no IL-17A^+^ CD4^+^ (Th17) T cells at this or earlier timepoints (Figure 1C). Th17 cells have been observed in humans, at times presumably long after initial infection (Ogongo et al., 2024; Sun et al., 2024; Nathan et al., 2021). We therefore tested whether Th17 cells arise at late timepoints after infection in mice. Mice continued to have a Th1 response up to day 170 post infection, with no increase in Th17 levels observed (Figure 1D and E). It was previously reported that infection with the virulent HN878 ‘Beijing’ strain of the L2 lineage resulted in induction of Th17 cells (Gopal et al., 2014). However, we did not observe a significant increase in IL-17A-producing T cells in murine lungs when we infected with a Beijing strain at 3 weeks post infection (Figure 1—figure supplement 1).

**Figure 1—figure supplement 1.**
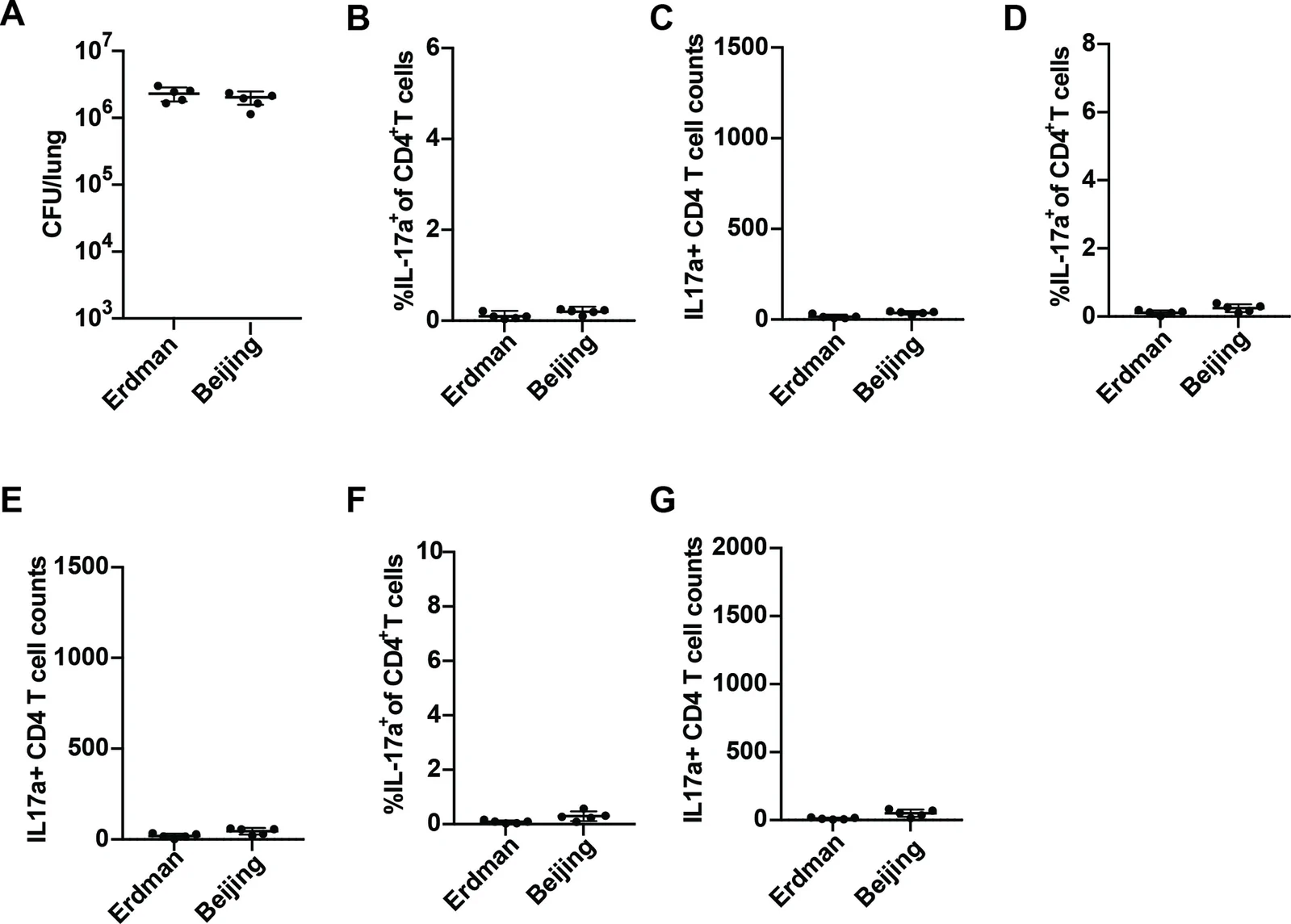
Mice were infected with either the Erdman or Beijing strain of Mtb via the aerosol route and evaluated at 21 dpi for (**A**) CFU in the lungs or (**B–G**) IL-17A^+^ CD4^+^ T cells in the lungs. (**B, C**) Unstimulated, (**D, E**) restimulated with antigen 85B peptide, and (**F, G**) restimulated with ESAT-6 peptide. Representative experiments of 2.

The route of bacterial infection can impact which T helper cell types are induced. Infection via the intranasal route has been shown to elicit Th17 cells in response to infection with *L. monocytogenes* and Group A *Streptococcus* (Pepper et al., 2010; Dileepan et al., 2011). We therefore tested whether the intranasal route of infection results in a Th17 response in the lungs of Mtb-infected mice. Mice were infected intranasally with ~100 CFU of WT Mtb into the lungs as measured on day 1 after infection. After 4 weeks of infection, mice infected by either route had comparable levels of bacteria in the lungs (Figure 1F). At this timepoint, we again observed only IFN-γ-producing CD4 T cells and very few IL-17A-producing CD4 T cells in the lungs (Figure 1G and H). Thus, the route of infection with Mtb does not impact the dominant Th1-biased response observed during primary infection of naïve mice with Mtb. We next investigated whether exogenous IL-17A treatment provides protection against WT Mtb infection during primary infection. WT mice aerosol infected with WT Mtb were intranasally administered with either PBS control or 1 μg recombinant murine IL-17A daily between days 11–20 post infection for a total of 10 doses (Figure 1I). WT mice intranasally (i.n.) administered 1 μg recombinant murine IL-17A daily were slightly more protected, with approximately threefold reduction in lung CFU compared to WT mice given vehicle control. However, we observed no effect of IL-17A when it was administered via the intraperitoneal (i.p.) route. Cytokines are known to have short half-lives in vivo. Fusing a cytokine to serum albumin can increase its half-life, permitting greater efficacy (Sleep et al., 2013). We therefore fused IL-17A to murine serum albumin (MSA-IL-17A) and administered this construct either i.p. or i.n. at equimolar quantities compared to free IL-17A administered dosages. We found that i.n. delivery of this construct was more effective than delivery of IL-17A, with approximately fivefold decrease in CFU observed in the lungs. In addition, fusion to albumin allowed us to observe efficacy with i.p. administration, resulting in an approximately twofold decrease in lung CFU. These results indicate that IL-17A can induce protective immunity to Mtb in the context of a primary infection, and that this may have potential as a therapeutic approach.

### *Tbx21*^–/–^ mice are protected by a robust Th17 response during early *M. tuberculosis* infection

**Figure 2.**
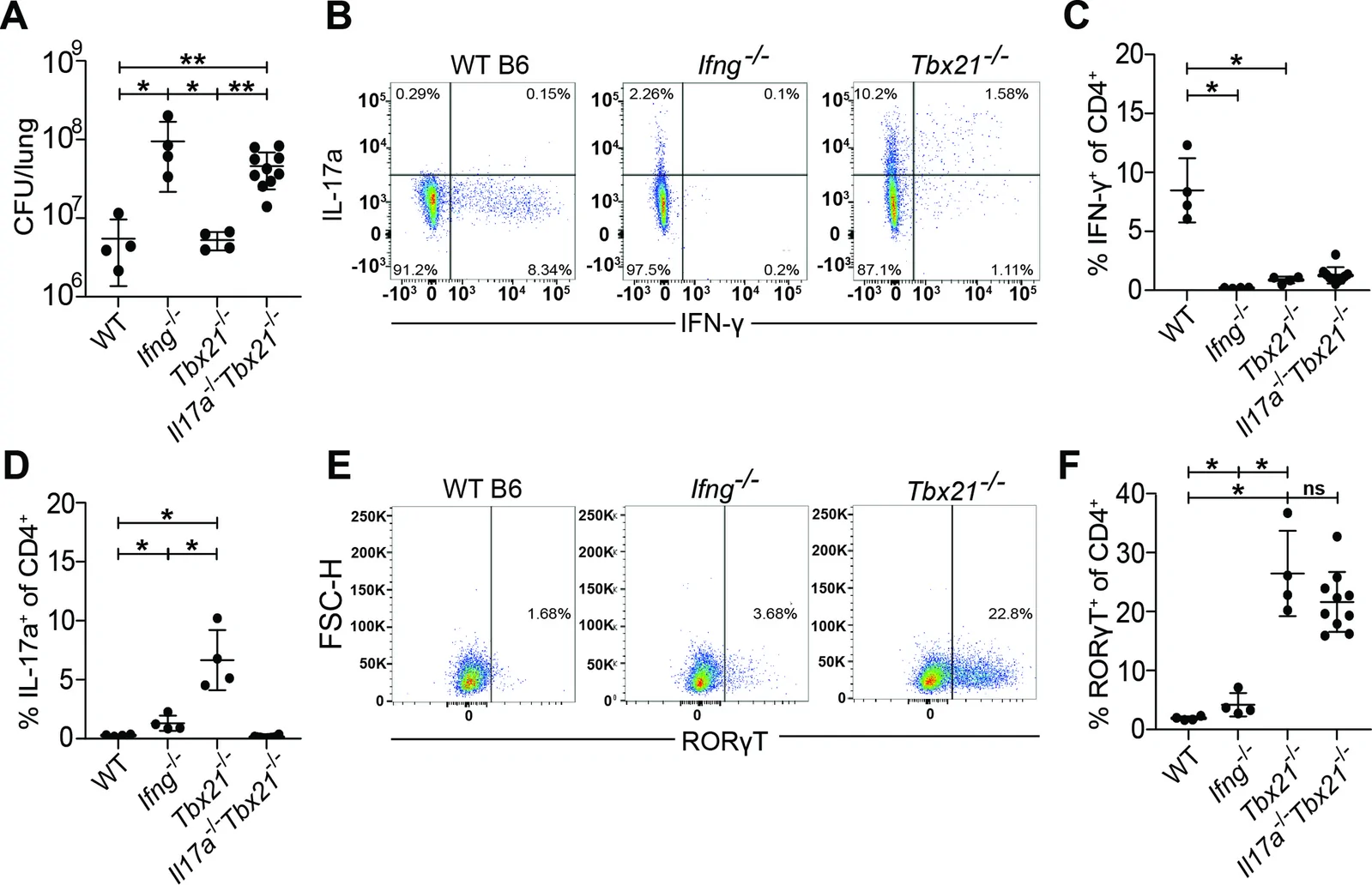
*Tbx21*^–/–^ mice are protected from susceptibility to Mtb infection by the emergence of Th17 cells. WT B6, *Ifng^–/–^*, *Tbx21^–/–^*, and *Il17a^–/–^Tbx21^–/–^* mice were aerosol infected with WT *M. tuberculosis* Erdman strain. *Tbx21^–/–^* and *Il17a^–/–^Tbx21^–/–^* mice were littermate-controlled. Mice were infected with Mtb and evaluated 21 days post infection for (**A**) CFU in the lungs of indicated genotypes or (**B–F**) cellular immune responses using flow cytometry. CD4^+^ T cells were gated on single, live, MHC-II^–^, Ly6G^–^, CD3^+^, γδTCR^–^, CD4^+^, CD8a^–^ cells for analysis of IFN-γ (**B, C**), IL-17 (**B, D**), and RORγT (**E, F**). Results in (A–F) are representative of three independent experiments. *p<0.05; **p<0.01 (unpaired nonparametric Mann–Whitney *U* test).

Because Th1 responses are known to inhibit Th17 responses (Lazarevic et al., 2011; Yeh et al., 2014), we next tested whether Th1 differentiation masks a protective Th17 response during Mtb infection. *Tbx21*, also referred to as Tbet, encodes the essential transcription factor that drives the Th1 lineage (Lazarevic et al., 2011). Interestingly, *Tbx21*^–/–^ mice lack Th1 T cells but are not as susceptible to infection with Mtb as *Ifng^–/–^* mice (Flynn et al., 1993; Sullivan et al., 2005). As expected, we found that *Ifng*^–/–^ mice were extremely susceptible to infection with Mtb; however, *Tbx21*^–/–^ had CFU in the lungs equivalent to wild-type mice at 21 days after infection (Figure 2A). Only WT mice had IFN-γ-producing CD4 T cells in the lungs at this timepoint (Figure 2B and C). Importantly, *Tbx21*^–/–^ mice exhibited a robust population of IL-17A-producing CD4 T cells at 3 weeks post infection, confirming that Tbet prevents Th17 differentiation during Mtb infection (Figure 2B and D). Importantly, while IFN-γ-deficient mice exhibited a modest increase in IL-17A-producing CD4 T cells (approximately fivefold) compared to WT B6 mice, *Tbx21-*deficient mice had a greater 25-fold induction of IL-17A-producing CD4 T cells. This demonstrates that IFN-γ production is not the sole mechanism by which Th1 T cells limit a Th17 response during Mtb infection (Figure 2D).

A previous study demonstrated that Tbet constrains effective T cell responses by promoting the differentiation of terminally differentiated nonprotective intravascular Th1 T cells (Sallin et al., 2017). This study also used antibody blockade of IL-17A in *Tbx21*-deficient mice to conclude that IL-17A provides minimal protection in this setting (Sallin et al., 2017). However, given that antibody blockade is limited by its short duration and possibly incomplete inhibition, we tested whether genetically ablating IL-17A production in *Tbx21*^–/–^ mice enhanced their susceptibility to Mtb infection. We therefore infected *Tbx21*^–/–^ and *Tbx21*^–/–^*Il17a*^–/–^ (*Il17a^icre/icre^* referred to as *Il17a*^–/–^) littermate-controlled mice with Mtb and measured CFU and T cell responses in the lungs after 3 weeks of infection. *Tbx21*^–/–^*Il17a*^–/–^ mice were highly susceptible to Mtb infection with a 10-fold increase in lung CFU compared to WT and *Tbx21*^–/–^ mice, comparable to the susceptibility observed in *Ifng^–/–^* mice (Figure 2A). *Tbx21*^–/–^*Il17a*^–/–^ mice had significantly fewer IFN-γ-producing (Figure 2C) and IL-17A-producing CD4 T cells (Figure 2D). Notably, *Tbx21^–/–^Il17a^–/–^* mice still generated Th17 cells, as evidenced by the presence of the Th17 master transcription factor RORγT (Lazarevic et al., 2011), despite their inability to produce IL-17A (Figure 2E and F). Together, these results indicate that IL-17A production by Th17 cells confers significant protection against Mtb infection in *Tbx21^–/–^* mice, at least during early stages of infection.

### *M. tuberculosis* utilizes ESX-1 and PDIM to limit Th17 response in mice

**Figure 3.**
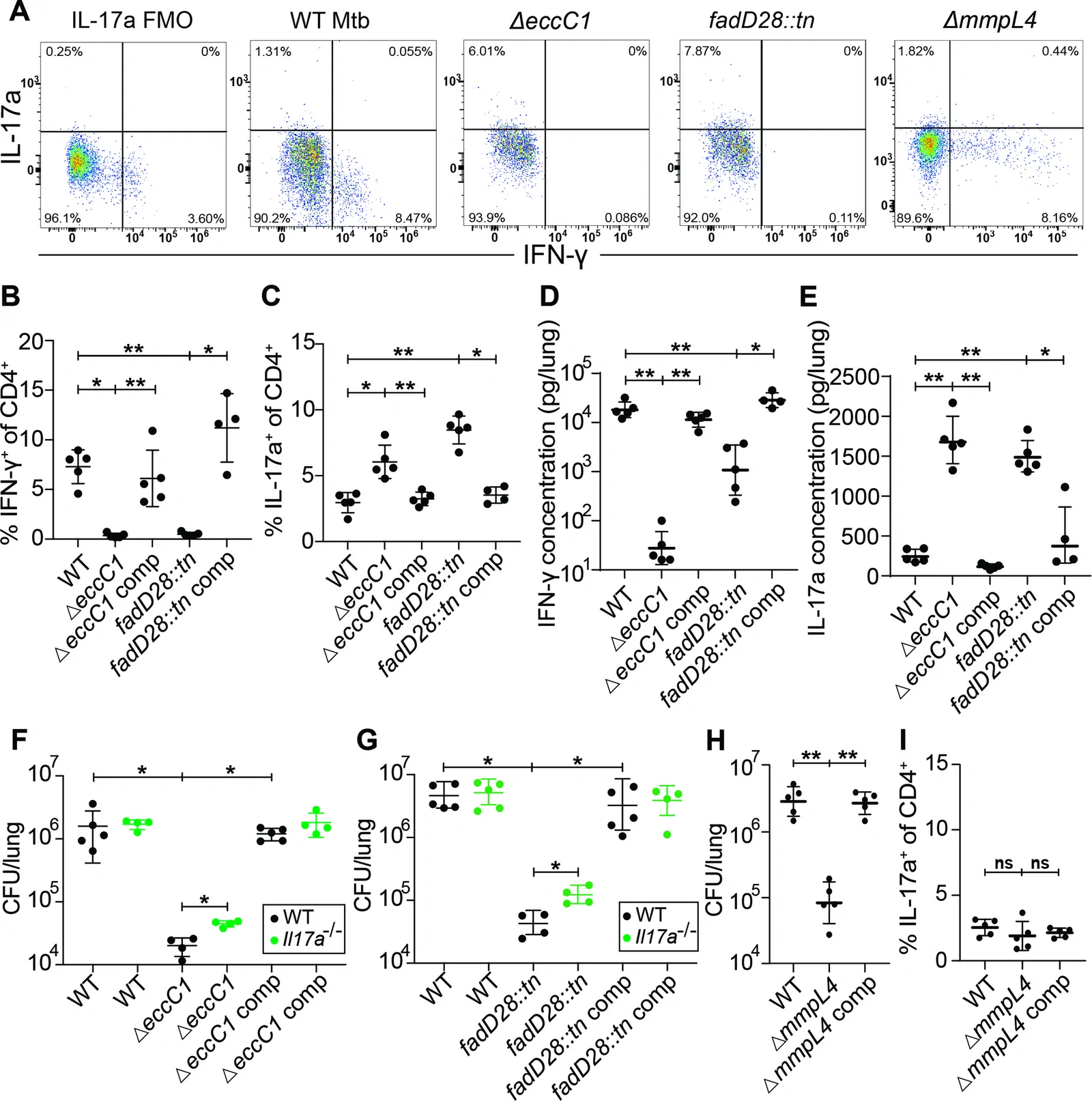
*M. tuberculosis* ESX-1 and PDIM virulence factors suppress Th17 response in mice. WT B6 mice were aerosol infected with indicated strains and evaluated at 21 days post infection. (**A**) Representative flow cytometry plots (cells gated on single, live, MHC-II^–^, Ly6G^–^, CD3^+^, γδTCR^–^, CD4^+^, CD8a^–^) of IFN-γ^+^ and IL-17A^+^ CD4^+^ T cell populations. Mice were sacrificed and evaluated with ESAT-6 peptide pool stimulated lung homogenates for (**B**) IFN-γ^+^ CD4^+^ T cells and (**C**) IL-17A^+^ CD4^+^ T cells by flow cytometry. Mice were sacrificed and lung homogenates were evaluated with BioLegend LEGENDplex for (**D**) IFN-γ and (**E**) IL-17A protein concentrations. (**F, G**) Littermate-controlled WT B6 and *Il17a^–/–^* mice were aerosol infected with indicated strains and sacrificed at 21 days post infection with lung homogenate plated for CFU. (**H, I**) Mice were infected with WT, *mmpl4* mutant, or complemented *mmpl4* mutant Mtb and evaluated for (**H**) day 21 CFU and (**I**) IL-17A^+^ CD4^+^ T cells by flow cytometry. Results are representative of two independent experiments. *p<0.05; **p<0.01 (Mann–Whitney *U* test).

We next examined whether Mtb utilizes virulence factors to alter the host T helper response. The ESX-1 secretion system and the complex lipid PDIM are both known to influence production of cytokines known to impact Th17 differentiation (Stanley et al., 2003; Stanley et al., 2007; Barczak et al., 2017; Hinman et al., 2021). Furthermore, these systems have been proposed to operate in the same virulence-promoting pathway (Barczak et al., 2017; Augenstreich et al., 2017). We therefore tested whether ESX-1 and PDIM might promote virulence in part by suppressing Th17 differentiation (Figure 3A). To test for a role of the ESX-1 secretion system, we infected mice with Δ*eccC1,* which lacks an ATPase required for ESX-1 function (ΔESX-1) (Crosskey et al., 2020; Figure 3—figure supplement 1). We also utilized a mutant with an inactivating transposon insertion in *fadD28*, a gene required for PDIM biosynthesis (*fadD28::tn*, PDIM-lacking) (Cox et al., 1999; Figure 3—figure supplement 1). As expected, ΔESX-1 and PDIM-lacking mutants were highly attenuated during infection at 3 weeks, exhibiting a 100-fold reduction in bacterial burden while complemented strains had similar CFU as WT Mtb (Figure 3—figure supplement 2). Interestingly, ESAT-6 peptide pool stimulation of lung cells in ΔESX-1 and PDIM-lacking Mtb-infected mice had an approximately fivefold reduction in %IFN-γ^+^ of CD4^+^ T cells (Th1s) (Figure 3B) and approximately twofold increase in %IL-17A^+^ of CD4^+^ T cells (Th17s) (Figure 3C) compared to complemented strains of either mutant or WT Mtb. Similar results were seen with Ag85b peptide pool stimulation (Figure 3—figure supplement 3). To validate flow cytometry results, we measured cytokine levels in lungs using BioLegend’s LEGENDplex kit. We found that ΔESX-1 and PDIM-lacking mutants induced less IFN-γ (Figure 3D) while also inducing ~10× higher levels of IL-17A (Figure 3E) in the lungs compared with WT and complemented Mtb strains (Figure 3D and E). We tested if these elevated levels of Th17 cells and IL-17A contribute to the attenuation of ESX-1 and PDIM mutants by infecting littermate-controlled WT B6 and *Il17a*^–/–^ mice with either WT, ΔESX-1, and complemented ΔESX-1 Mtb, or WT, PDIM-lacking, and complemented PDIM-lacking Mtb. Under the conditions where Th17s are highly induced, mice infected with either ΔESX-1 or PDIM-lacking Mtb, the *Il17a*^–/–^ mice had approximately three- to fivefold higher CFU than WT mice (Figure 3F and G). These results indicate that the induction of Th17s is not dependent on the attenuation of Mtb in general, but instead Mtb utilizes ESX-1 and PDIM to suppress the induction of a Th17 response that enhances protection against Mtb infection.

**Figure 3—figure supplement 1.**
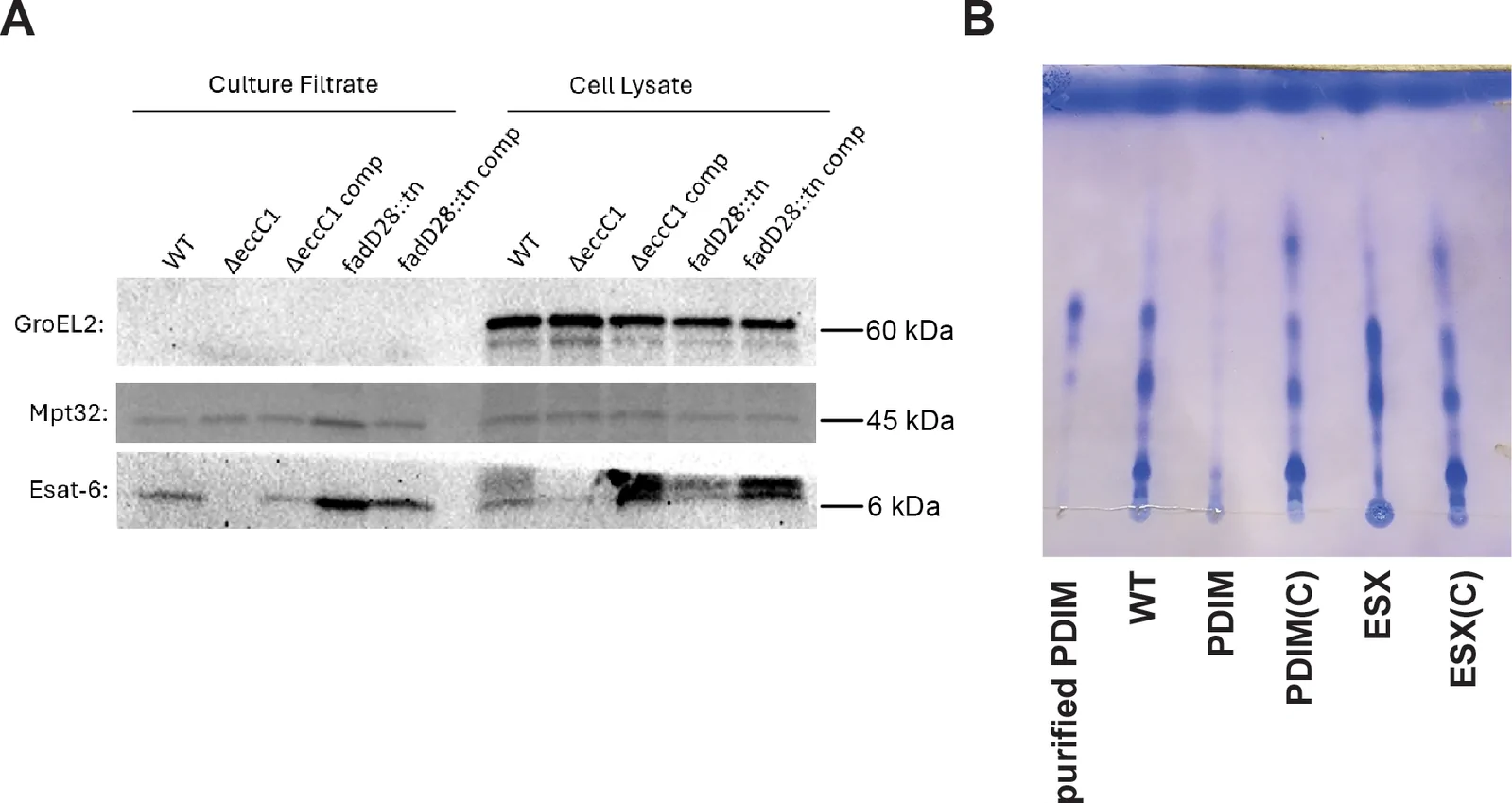
Mtb Erdman PDIM mutants secrete ESAT-6, and ESX-1 mutants produce PDIM. (**A**) WT, Δ*eccC1*, complemented Δ*eccC1::eccC1* (Δ*eccC1* comp), *fadD28::tn*, or *fadD28::tn* +p*fadD28* (*fadD28*::tn comp) *M. tuberculosis* Erdman strain were cultured in Sauton’s complete media without Tween 80 for 5 days. Cell lysate and culture supernatant were processed through SDS-PAGE and western blot analysis. Anti-ESAT-6 antibody was used for detecting ESAT-6. Anti-Mtb GroEL2 antibody (BEI Resources NR-13657) was used as a loading control for the cell lysate fraction. Anti-Mtb Mpt32 antibody (BEI Resources NR-13807) was used as a loading control for the culture filtrate. Results shown are from one experiment representative of two independent experiments. (**B**) WT, Δ*eccC1* (ESX), complemented Δ*eccC1::eccC1* (ESX(C)), *fadD28::tn* (PDIM), or *fadD28::tn* +p*fadD28* (PDIM(C)) *M. tuberculosis* Erdman strain were cultured in 7H9 media. Outer leaflet of mycomembrane was removed via hexanes wash, concentrated and separated on TLC plate with 98:2 petroleum ether:acetone mobile phase. TLC plate was stained with 0.2% Coomassie blue in 20% methanol. Results shown are from one experiment representative of two independent experiments. Figure 3—figure supplement 1—source data 1.Unedited western blot images. Figure 3—figure supplement 1—source data 2.Edited document of entire western blot images.

**Figure 3—figure supplement 2.**
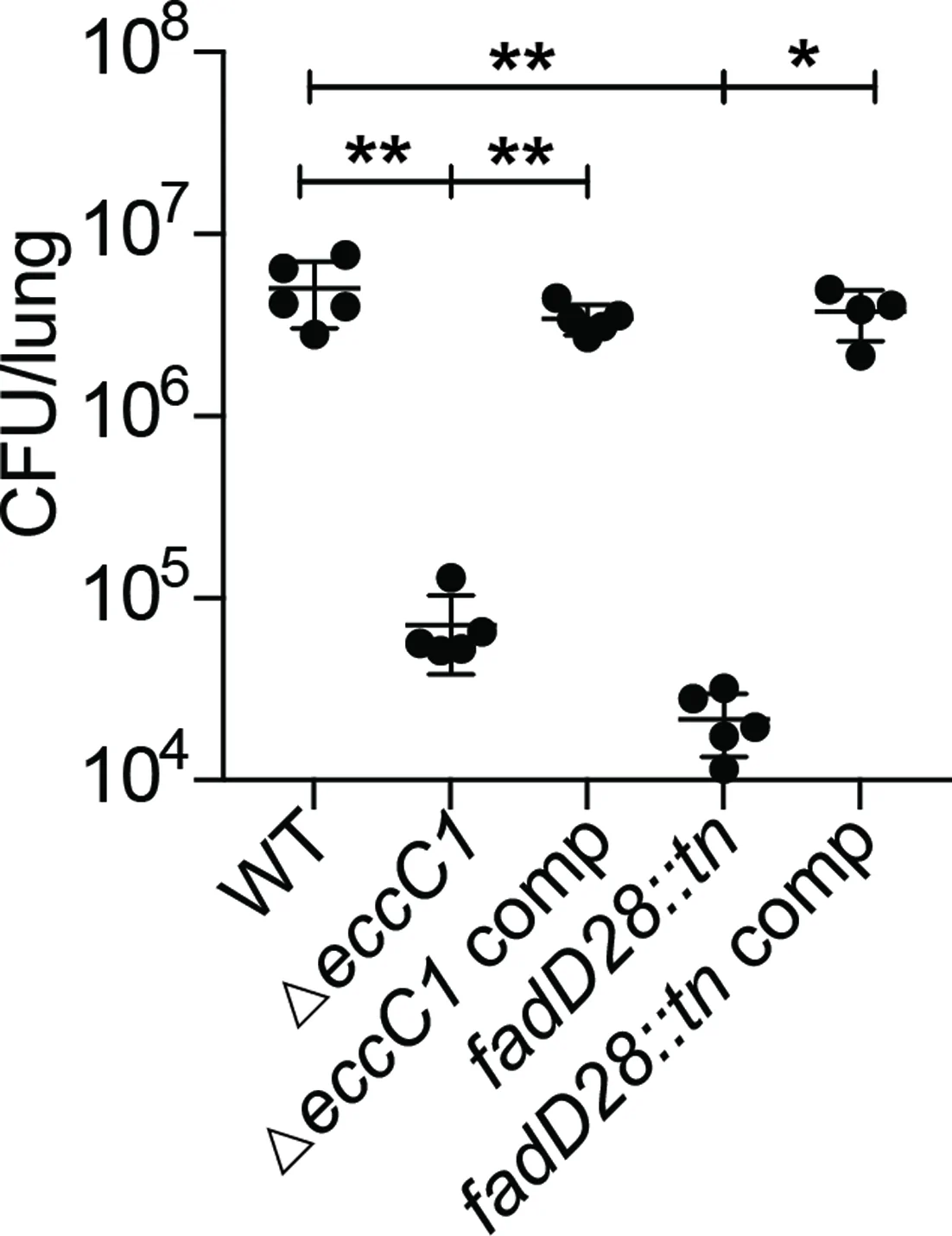
Attenuation of ESX-1 and PDIM mutants at 21 days post infection. WT B6 mice were aerosol infected with either WT, Δ*eccC1,* Δ*eccC1::eccC1*, *fadD28::tn*, or *fadD28::tn +* p*fadD28 M. tuberculosis* Erdman strain. 21 days post infection, mice were sacrificed, and CFU were enumerated from lung lysates.

**Figure 3—figure supplement 3.**
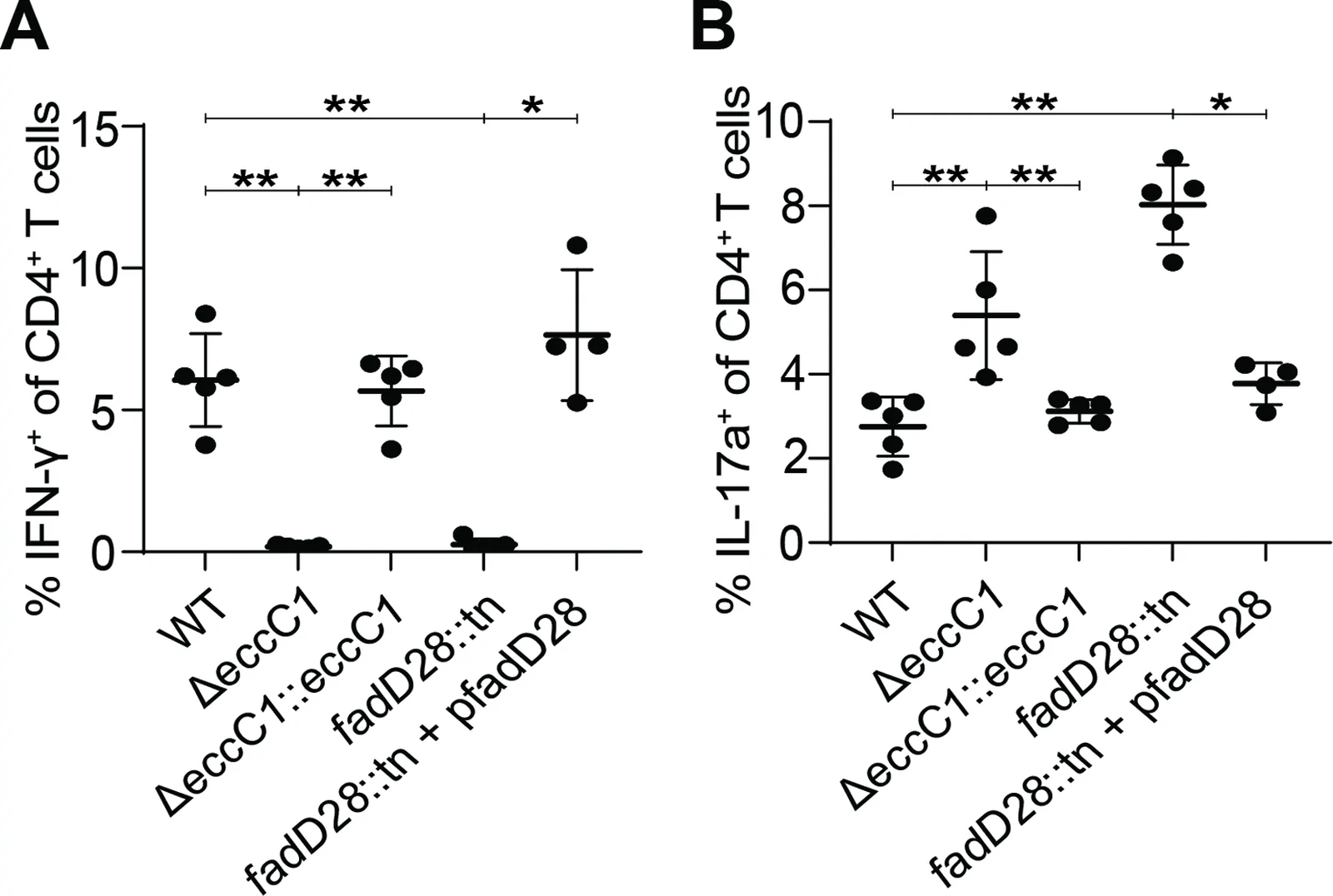
Antigen 85b stim resembles ESAT-6 stimulation for IFN-γ and IL-17A staining of CD4^+^ T cells. As in Figure 3, WT B6 mice were aerosol infected with either WT, Δ*eccC1,* Δ*eccC1::eccC1*, *fadD28::tn*, or *fadD28::tn* + p*fadD28 M. tuberculosis* Erdman strain. 21 days post infection, mice were sacrificed, lung single cell homogenates were stimulated with Antigen 85b peptide pool and measured for lung (**A**) IFN-γ^+^ CD4^+^ T cells and (**B**) IL-17A^+^ CD4^+^ T cells by flow cytometry. Results in (A) and (B) are representative of two independent experiments. *p<0.05; **p<0.01 (unpaired nonparametric Mann–Whitney *U* test).

**Figure 3—figure supplement 4.**
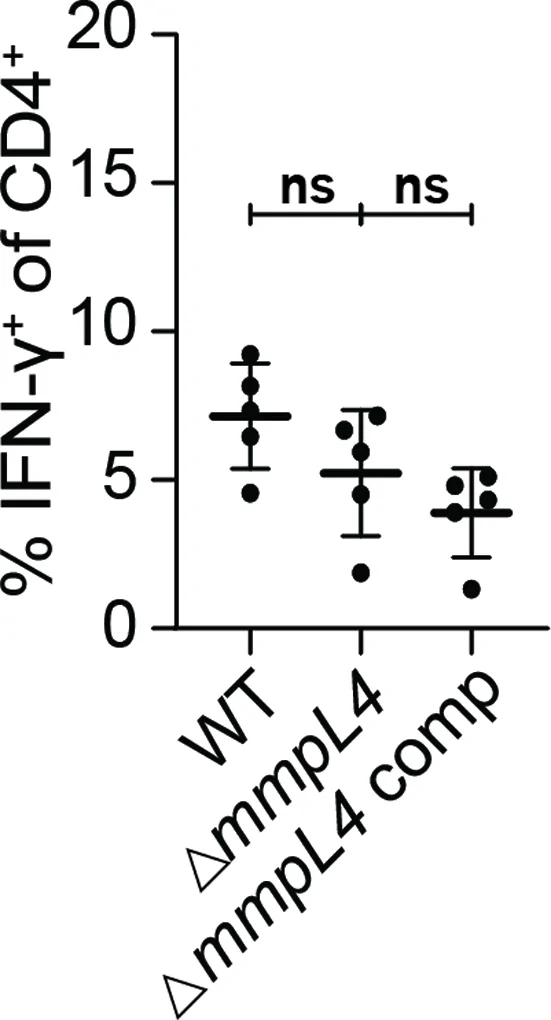
Δ*mmpL4* Mtb induces similar IFN-γ^+^ CD4^+^ T cells as WT or complemented Mtb. Mice were aerosol infected with WT, Δ*mmpL4* mutant, or complemented Δ*mmpL4* Mtb Erdman strain and evaluated for IFN-γ^+^ CD4^+^ T cells after restimulation with ESAT-6 peptide by flow cytometry. Results are representative of two independent experiments. *p<0.05; **p<0.01 (unpaired nonparametric Mann–Whitney *U* test).

To ensure that the increase in Th17s and IL-17A was not simply a consequence of infection with any attenuated Mtb mutant, we evaluated responses during infection with MmpL4-deficient (Δ*mmpL4*) bacteria. MmpL4 is a transmembrane transporter with proposed siderophore secretion activity (Wells et al., 2013) with no known association with the function of ESX-1 or secretion of PDIM (Cox et al., 1999). Δ*mmpL4* Mtb-infected mice had ~1.5 logs lower lung CFU than WT or complemented Mtb-infected mice (Figure 3H), a similar degree of attenuation as we observed with ESX-1 and PDIM mutants (Figure 3—figure supplement 2). However, the Δ*mmpL4* Mtb-infected mice induced a Th1 response that was indistinguishable from WT and complemented Mtb-infected mice (Figure 3—figure supplement 4) while not inducing greater numbers of Th17s compared to WT or complemented Mtb-infected mice (Figure 3I). Thus, the increase in Th17s we observed results from an ESX-1/PDIM specific mechanism.

### Type I interferons boost Th1 induction and do not impact Th17 induction

**Figure 4.**
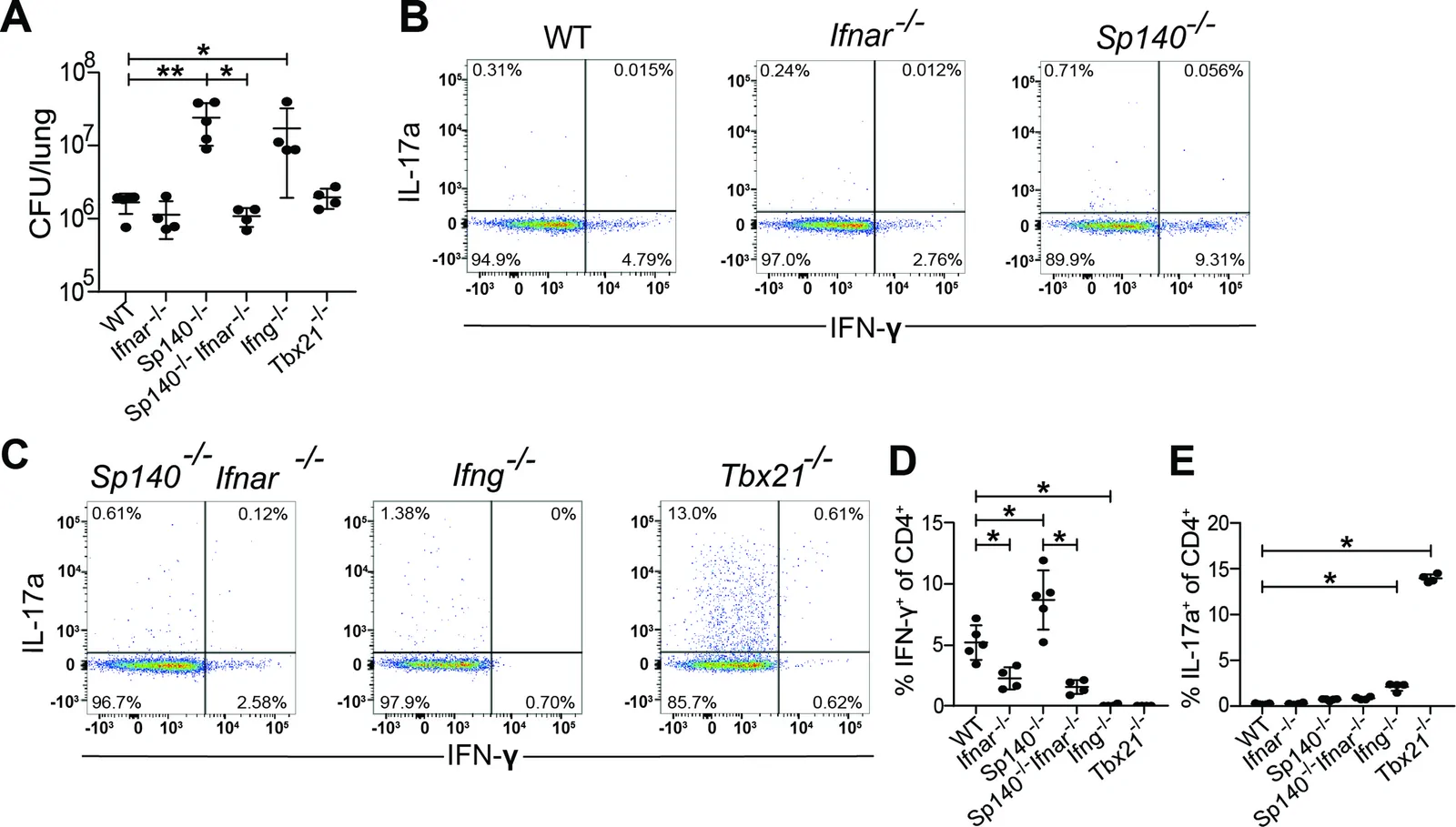
Type I interferon signaling boosts Th1 induction and does not impact Th17 induction. WT B6, *Ifnar^–/–^*, *Sp140^–/–^*, *Sp140^–/–^Ifnar^–/–^*, *Ifng^–/–^*, and *Tbx21^–/–^* mice were aerosol infected with WT *M. tuberculosis* Erdman strain. (**B, C**) Representative flow cytometry plots (cells gated on single, live, MHC-II^–^, Ly6G^–^, CD3^+^, γδTCR^–^, CD4^+^, CD8a^–^) of IFN-γ^+^ and IL-17A^+^ CD4^+^ T cells of these populations. 21 days post infection, lung homogenates were measured for (**A**) CFU and analyzed via flow cytometry for (**D**) IFN-γ^+^ CD4^+^ T cells and (**E**) IL-17A^+^ CD4^+^ T cells. Results in (A), (D), and (E) are representative of two independent experiments. *p<0.05; **p<0.01 (unpaired nonparametric Mann–Whitney *U* test).

As ESX-1 and PDIM are responsible for the host type I interferon response to Mtb infection (Stanley et al., 2007; Barczak et al., 2017), and type I IFN is known to suppress Th17 responses (Ramgolam et al., 2009), we investigated the impact of type I interferon signaling on Th17 induction during Mtb infection. We examined the effect of type I IFN both in WT B6 mice and in *Sp140^–/–^* mice that are highly susceptible to infection because of an excessive type I IFN response to infection (Ji et al., 2021). To abrogate type I IFN in each background, we used mice deficient for the type I IFN receptor IFNAR. WT, *Ifnar1*^–/–^, *Sp140*^–/–^, *Sp140*^–/–^*Ifnar1*^–/–^, *Ifng*^–/–^, and *Tbx21*^–/–^ mice were aerosol infected with WT Mtb. As expected, both *Sp140*^–/–^ and *Ifng*^–/–^ mice had up to 10-fold higher CFU than WT mice while *Ifnar1*^–/–^, *Sp140*^–/–^*Ifnar1*^–/–^, and *Tbx21*^–/–^ mice were as resistant as WT mice at 3 weeks post infection (Figure 4A). *Sp140*^–/–^ mice had an almost twofold increase in %IFN-γ^+^ of CD4^+^ (Th1) T cells compared to WT mice, *Ifnar1*^–/–^ had a twofold reduction in %IFN-γ^+^ of CD4^+^ (Th1) T cells compared to WT mice, *Sp140*^–/–^*Ifnar1*^–/–^ mice had a slightly stronger fourfold reduction in %IFN-γ^+^ of CD4^+^ (Th1) T cells compared to *Sp140*^–/–^ mice (Figure 4B–D). Importantly, deletion of IFNAR had no impact on Th17 induction in either WT or *Sp140*^–/–^ mice, although we observed the expected increase in Th17 cells in *Tbx21*-deficient animals (Figure 4E). These results indicate that type I interferon signaling only impacts the induction of Th1s and does not influence Th17 induction, suggesting that induction of type I IFN by ESX-1/PDIM is not the mechanism by which these virulence factors suppress Th17 differentiation.

### ESX-1 and PDIM alter conventional dendritic cell cytokine signaling in the mediastinal lymph node

**Figure 5.**
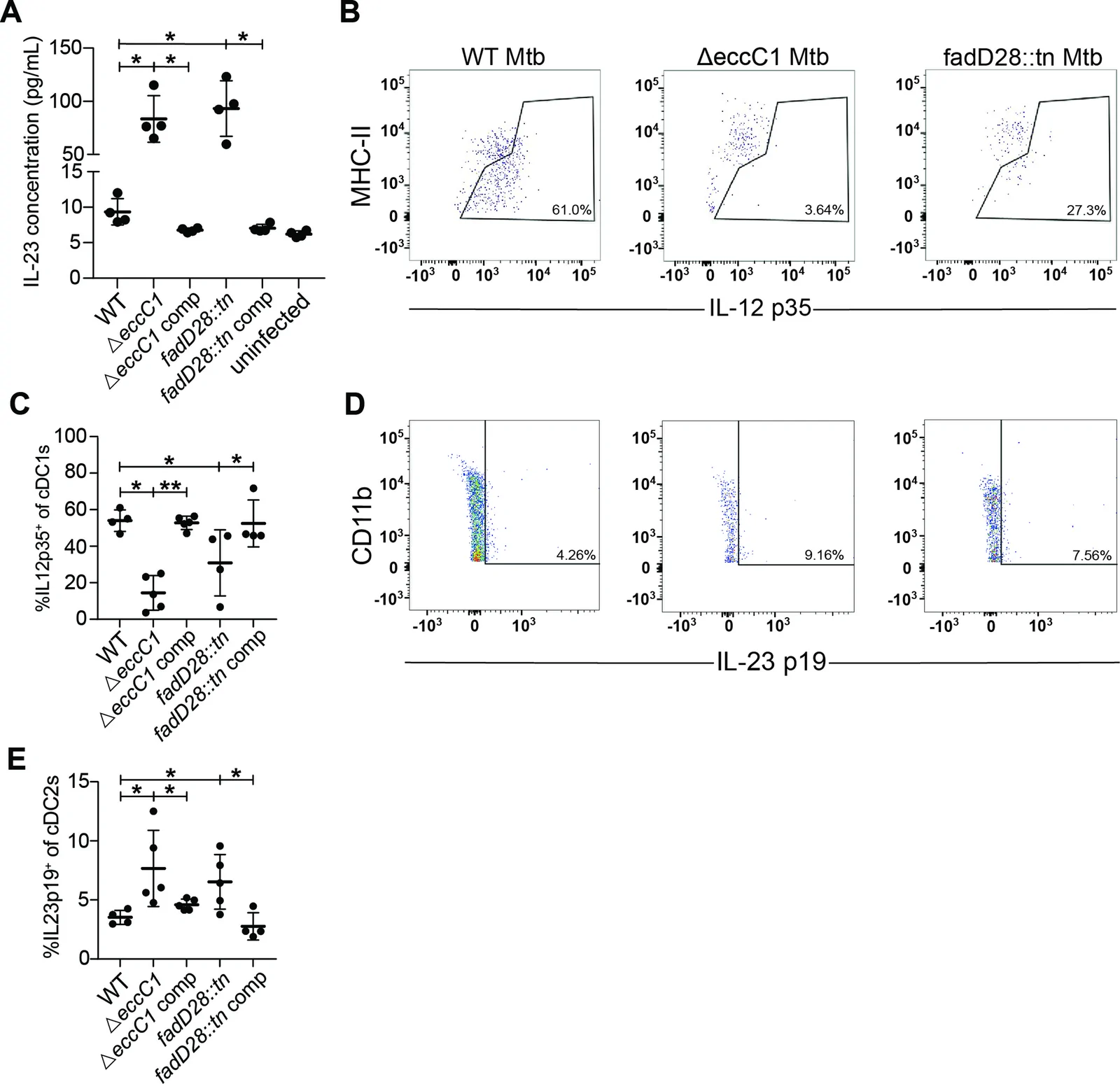
ESX-1 and PDIM alter conventional dendritic cell cytokine signaling in the mediastinal lymph node. WT B6 bone marrow-derived macrophages were infected with designated Mtb Erdman strains at MOI = 10 or uninfected for 24 hours with (**A**) IL-23 ELISA taken from cultured supernatants. WT B6 mice were aerosol infected with indicated strains and analyzed at 21 days post infection. (**C, E**) Flow cytometry schematic for gating of (**B**) cDC1 (gated on single, live, CD3^–^, CD19^–^, CD11c^+^, MHC-II^+^, XCR1^+^, CD11b^–^) and (**D**) cDC2 (gated on single, live, CD3^–^, CD19^–^, CD11c^+^, MHC-II^+^, XCR1^–^, CD11b^+^) populations of mediastinal lymph nodes with representative flow cytometry plots of IL-12 p35^+^ and IL-23 p19^+^ gating of cDC1 and cDC2 populations respectively. 21 days post infection, mediastinal lymph nodes from WT B6 mice were aerosol infected with either WT, Δ*eccC1* (ΔESX-1), complemented Δ*eccC1*::*eccC1*, transposon-inserted *fadD28*::tn (PDIM-lacking), or complemented transposon-inserted *fadD28*::tn + p*fadD28 M. tuberculosis* Erdman strain were extracted and analyzed for IL-12 p35^+^ cDC1s (**C**) and IL-23 p19^+^ cDC2s (**E**) by flow cytometry. Results in (A), (C), and (E) are representative of two independent experiments. *p<0.05; **p<0.01 (unpaired nonparametric Mann–Whitney *U* test).

Previous work suggested that ESX-1 mutants fail to suppress expression by macrophages of IL-12 p40, a subunit shared between IL-12 and IL-23 (Stanley et al., 2003). Because IL-23 promotes Th17 responses, and ESX-1 and PDIM are responsible for the lack of a Th17 response during Mtb infection, we next investigated if ESX-1 and PDIM suppress IL-23 production in bone marrow-derived macrophages (BMDMs) during in vitro infection. ESX-1 and PDIM-deficient strains both elicited a strong IL-23 response measured using IL-23 ELISA (Figure 5A). We then investigated if ESX-1 and PDIM suppress IL-23 production by dendritic cells during in vivo infection in the draining (mediastinal) lymph nodes. We infected mice with ~100 CFU via the aerosol route and harvested mediastinal lymph nodes at 21 days post infection. We examined IL-12 and IL-23 production by conventional dendritic cells using ICS. To clearly distinguish between levels of these two cytokines, we used ICS for the cytokine-specific subunits: IL-12 p35 and IL-23 p19. First, we found that type 1 conventional dendritic cells (cDC1s), known to be specialized for IL-12 production, produce robust levels of IL-12 p35 during infection with WT Mtb (Figure 5B and C). However, both ESX-1 and PDIM-deficient strains elicited significantly lower levels of IL-12 p35 from these cells (Figure 5B and C), consistent with the lower bacterial burdens in the lungs of mice infected with these mutants (Figure 3—figure supplement 2). During infection with wild-type bacteria, we observed very little IL-23 p19 production by type 2 conventional dendritic cells (cDC2s) in the draining mediastinal lymph node (Figure 5D and E). Importantly, ESX-1 and PDIM-lacking Mtb infection resulted in an increase in the percentage of cDC2s that produce IL-23 p19 in mediastinal LNs (Figure 5D and E). We saw very little to no IL-23 p19 expression in cDC1s and IL-12 p35 expression in cDC2s with no differences between WT or ESX-1 and PDIM-deficient strains (Figure 5—figure supplement 1). These results indicate that the ESX-1 and PDIM virulence factors impact T helper cell subsets observed in the lungs by promoting IL-12, a Th1-polarizing cytokine, and limiting IL-23, a Th17-polarizing cytokine, expression in cDC1s and cDC2s respectively in the draining mediastinal lymph node, which may impact naïve T cell differentiation.

**Figure 5—figure supplement 1.**
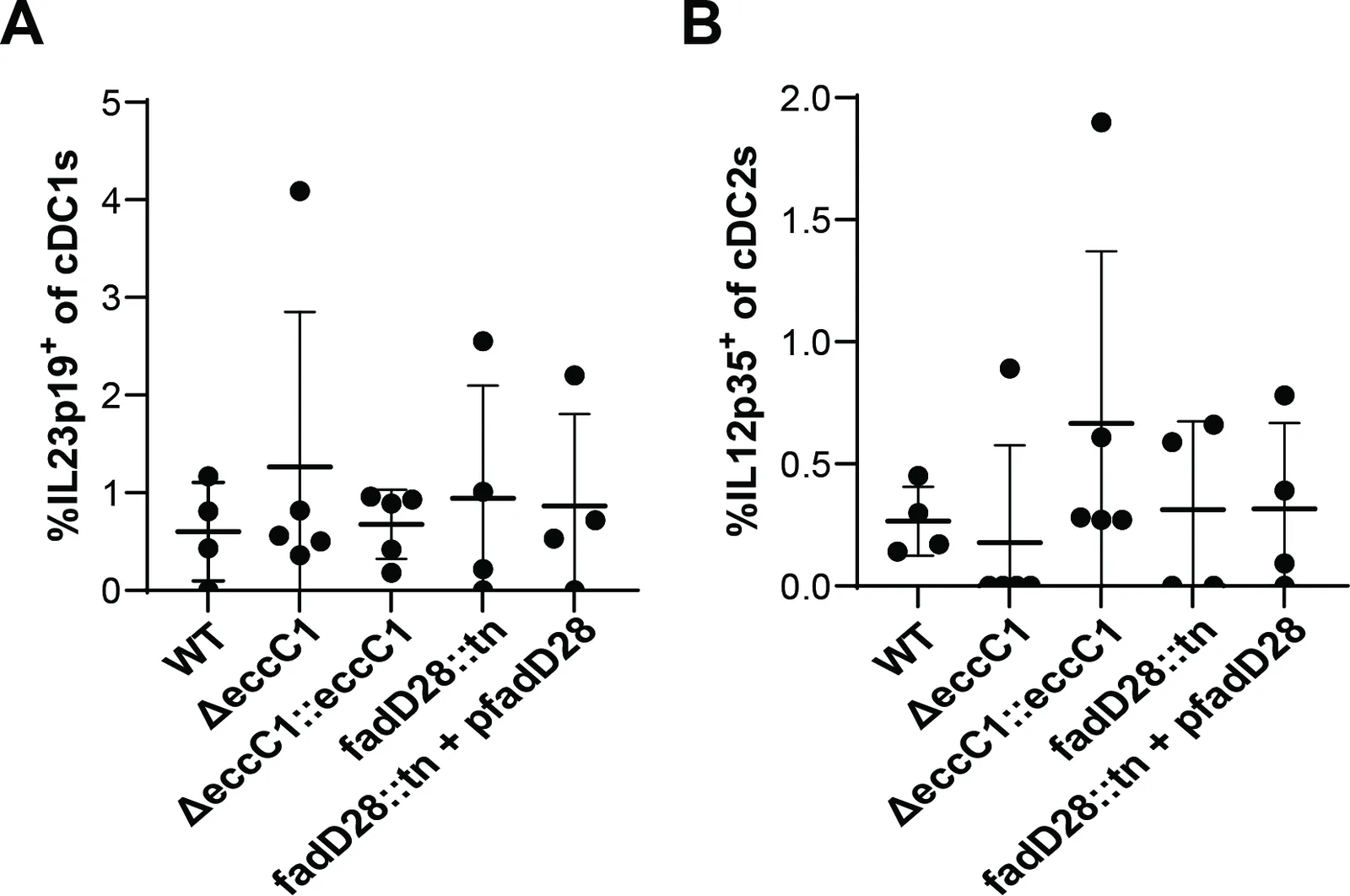
There is little to no IL-23 p19 expression in cDC1s and IL-12 p35 expression in cDC2s during Mtb infection. As in Figure 5, WT B6 mice were aerosol infected with WT, Δ*eccC1,* Δ*eccC1::eccC1*, *fadD28::tn*, or *fadD28::tn* + p*fadD28 M. tuberculosis* Erdman strain. 21 days post infection, mice were sacrificed, mediastinal lymph nodes were extracted and processed for ICS. Analysis of (**A**) IL-23 p19^+^ cDC1s and (**B**) IL-12 p35^+^ cDC2s by flow cytometry. Results in (A) and (B) are representative of two independent experiments (unpaired nonparametric Mann–Whitney *U* test).

## Discussion

The rise of multidrug-resistant and extensively drug-resistant strains, combined with a limited pipeline of new drugs, underscores the urgent need for effective vaccines. Achieving the World Health Organization/STOP TB Partnership’s target of TB elimination by 2050 will not be possible without a highly efficacious vaccine (Dye et al., 2013). Unlike most successful vaccines, which protect through neutralizing antibodies, TB immunity primarily depends on T-cell-mediated responses, for which no clear vaccine strategy exists. Defining protective immune mechanisms is therefore critical—not only for guiding vaccine design but also for advancing immune-based therapies. Equally important is understanding how *M. tuberculosis* evades or suppresses these responses, as such insights can inform both vaccine development and therapeutic interventions.

We sought to understand why Mtb infection does not elicit Th17 production robustly during primary infection. We found that this is independent of duration and route of infection but instead results from an overly robust Th1 response, which suppresses Th17 development, and upon the virulence factors ESX-1 and PDIM. Perhaps Mtb uses ESX-1 and PDIM to allow other virulence factors like Hip1 to access the host cytosol, alter host cellular signaling, and impair dendritic cell expression of IL23 p19, CD40, Jagged, and DLL4 shown to be important for dendritic cell-mediated differentiation of naïve T helper cells into Th17s (Madan-Lala et al., 2011; Sia et al., 2017; Enriquez et al., 2022; Khader et al., 2005). It has been long established that Th1 cells are important for control of Mtb infection, yet there have also been hints in the literature that Th1 cells are not simply protective. Firstly, excessive IFN-γ production is harmful, driving tissue destruction in the lungs (Sakai et al., 2016), which may benefit bacteria by promoting necrosis and transmission. Interestingly, the majority of T cell antigens are highly conserved across Mtb (Comas et al., 2010), suggesting that dominant Th1 cell responses may confer some benefit to Mtb. Indeed, a recent study suggested that while common antigens are likely to drive Th1 differentiation, rare variable antigens are more likely to drive Th17 differentiation in Mtb-infected patients (Ogongo et al., 2026). Thus, it is feasible that Mtb promotes excessive Th1 differentiation in part to prevent potentially protective Th17 cells from emerging.

Given that ESX-1 and PDIM mutants elicit type I IFN, and type I IFN suppresses Th17 cells, it is perhaps surprising that type I IFN does not explain the suppression of Th17 we observe during early infection. Type I IFN is clearly detrimental to Mtb infection, as deletion of IFNAR rescues the susceptibility of mice that exhibit type I IFN-driven disease (Stanley et al., 2007; Ji et al., 2021). However, type I IFN is known to have many immunosuppressive functions that could explain its detrimental role in Mtb infection (McNab et al., 2015). Many cytokines are known to participate in the differentiation of Th17 cells, including TGF-β, IL-6, and IL-23. Importantly, IL-23 is essential for maintaining the Th17 lineage, while TGF-β and IL-6 are critical for its induction. The crucial role of IL-23 differentiation and maintenance of Th17 cells in vivo is well established (Gaffen et al., 2014; Stritesky et al., 2008). IL-23 supports IL-17 production by upregulating IL-17 itself, RORγt, and other pro-inflammatory cytokines (Krueger et al., 2024). Our in vivo data demonstrating increased IL-23 production by dendritic cells in ESX-1 mutant infections is consistent with the old observation that ESX-1 suppresses IL-12 p40 in vitro. Future work will focus on elucidating the mechanism of Mtb suppression of IL-23. It is also possible that ESX-1/PDIM suppress Th17 differentiation by promoting Th1 differentiation; however, we do not have data that supports this model at this time. Finally, the work in this study adds to the growing evidence that ESX-1 and PDIM are phenotypically linked. Unlike what has been demonstrated in the H37Rv strain (Barczak et al., 2017), we do not find that PDIM mutants have a defect in ESX-1 function or that ESX-1 mutants have any changes in PDIM production or secretion. This could be a strain-specific difference, especially as it is known that H37Rv has a unique point mutation that causes downregulated ESX-1 function relative to Erdman and most clinical strains (Solans et al., 2014). In the future, it will be important to establish how ESX-1 and PDIM function are linked functionally. Secretion of the ESX-1 substrate ESAT-6 is generally used as a proxy for ESX-1 function. It is possible that PDIM loss has more subtle effects on ESX-1 function that are not reflected by ESAT-6 secretion levels.

Because primary infection does not reliably elicit Th17 T cells, it has been difficult to study the function of these cells. A method for reliably inducing Th17s in mice during infection would facilitate studying the role of Th17-mediated protection. We show here that mice lacking *Tbx21* respond to WT Mtb infection with a Th17 response and provide protection mediated by IL-17A during early infection. Thus, *Tbx21*^–/–^ mice can be used to interrogate mechanisms underlying Th17-mediated protection during primary Mtb infection. High Th17 plasticity permits Th17s to express a wide variety of T helper subset profiles that can range from pro-inflammatory Th17.1s that co-express IFN-γ and IL-17A to anti-inflammatory Tr1-like Th17s that co-express IL-10 and IL-17A (Stadhouders et al., 2018). Moving forward, it will be important to delineate in depth which Th17 subsets and which mechanisms downstream of IL-17 receptor signaling are important for controlling Mtb infection. Importantly, we show that exogenous administration of recombinant IL-17A leads to a decrease in CFU in Mtb-infected lungs by up to ~5×. Our data suggests that a limiting factor for efficacy of recombinant IL-17A is its short half-life. Future efforts focused on methods for improving the stability of this cytokine in vivo could lead to more dramatic reductions in bacterial burdens.

The live vaccine strain Bacille Calmette–Guerin (BCG) was developed by extensive laboratory passage of *M. bovis*, resulting in attenuation of virulence (Mahairas et al., 1996; Hsu et al., 2003). This attenuation makes BCG a very safe vaccine. However, although BCG is effective in preventing severe tuberculosis in children, it is not sufficiently effective against adult pulmonary tuberculosis to make a significant impact on TB cases worldwide. A cause of the reduced virulence of BCG is the loss of multiple genes associated with the ESX-1 type VII secretion system. To create a live vaccine that is safe yet more effective than BCG, scientists engineered MTBVAC, a vaccine candidate currently in phase 2b trials. Unlike BCG, MTBVAC is derived from Mtb. MTBVAC is attenuated by mutation of the ESX-1 secretion system and a mutation that eliminates PDIM (Arbues et al., 2013). Our insights into how ESX-1 and PDIM suppress effective immune responses could promote understanding of how MTBVAC provides protective immunity. Indeed, MTBVAC has been demonstrated to elicit mucosal-homing-marker expressing Th17s ideal for better control of Mtb infection (Dijkman et al., 2021). In addition, understanding the role and function of Th17 cells more generally can help promote development of the next generation of effective Mtb vaccine candidates.

## Materials and methods

### Bacterial culture

*M. tuberculosis* strain Erdman was grown in Middlebrook 7H9 liquid medium supplemented with 10% oleic acid, albumin, dextrose, and catalase (OADC) (BD 212351), 0.4% glycerol, and 0.05% Tween 80 or on solid 7H10 agar plates supplemented with 10% OADC and 0.4% glycerol. Frozen stocks of *M. tuberculosis* were made from a single culture and used for all experiments.

### Mice

C57BL/6J (no. 000664), *Ifng*^–/–^ (B6.129S7-*Ifng*^tm1Ts^/J, no. 002287), *Tbx21*^–/–^ (B6.129S6-*Tbx21*^tm1Glm^/J, no. 004648), and *Il17a*^icre/icre^ (*Il17a*^–/–^, B6.129(SJL)-*Il17a*^tm1.1(icre)Stck^/J, no. 035717) mice were obtained from the Jackson Laboratory and bred in-house. *Ifnar1*^–/–^, *Sp140*^–/–^, and *Sp140*^–/–^*Ifnar1*^–/–^ mice were obtained from the Vance laboratory (UC Berkeley) and bred in-house. Mice were age matched for all experiments.

### Aerosol challenge experiments with *M. tuberculosis*

Mice between the age of 8–12 weeks old were infected via the aerosol route with *M. tuberculosis* strain Erdman using a nebulizer and full-body inhalation exposure system (Glas-Col, Terre Haute, IN). A total of 9 mL of diluted aerosol stock in MilliQ water was loaded into the nebulizer to deliver 30–100 bacteria per mouse as measured by whole-lung CFU 1 day post infection.

### Intranasal challenge experiments with *M. tuberculosis*

Mice between the age of 8–12 weeks old were infected with 40 uL of *M. tuberculosis* strain Erdman diluted in PBS administration for mice to inhale 30–100 bacteria per mouse as measured by whole lung CFU 1 day post infection.

### Flow cytometry

Whole lungs were homogenized using GentleMACS C tubes (Miltenyi Biotec 130-093-237) in complete RPMI-1640 media supplemented with 10% FBS, 1 mM sodium pyruvate (Sigma-Aldrich S8636), 0.01 mM HEPES, 1% GlutaMAX (Thermo Scientific 35050061), 1% non-essential amino acids (Gibco 11140-050), and 55 μM 2-mercaptoethanol (Gibco 21985-023) containing Liberase TM (Sigma-Aldrich 05401119001) and DNase I (Sigma-Aldrich 11284932001). Cells were strained through 70-micron strainer (Corning 352350). Cells were treated with protein transport inhibitor cocktail mix (Invitrogen 00-4980-03) and stimulated with vehicle control, Mtb Ag85b peptide pool (Peptides&elephants LB02088), Mtb esat6 peptide pool (Peptides&elephants LB02113), or PMA and ionomycin (Invitrogen 00-4970-93) for 5 hours at 37°C, 5% CO_2_. Cells were stained with live/dead stain (Thermo Fisher L23101), Fc block (BioLegend 101320), and surface stained with antibodies for CD3, Ly6G, CD4 (BD 740268, 551460, and 569182, respectively) (RRID:AB_2687927, AB_394207, and AB_3684848, respectively), MHC-II, pan-γδTCR, and CD8a (BioLegend 107605, 118124, and 100714, respectively) (RRID:AB_313320, AB_11204423, and AB_312753, respectively) in Brilliant stain buffer (BD 566349). Cells were fixed and permeabilized with Foxp3 staining kit (Invitrogen 00-5523-00) before intracellular staining for RORγT, Foxp3 (BD 564723 and 562996, respectively) (RRID:AB_2738916 and AB_2737940, respectively), Tbet, IFN-γ, and IL-17A (BioLegend 644817, 505814, and 506903, respectively) (RRID:AB_11219388, AB_493314, and AB_315463, respectively). Data was collected using a BD Symphony A3 flow cytometer (BD) and analyzed using FlowJo Software (BD).

Mediastinal lymph nodes were homogenized using complete RPMI-1640 containing Liberase TM (Sigma-Aldrich 05401119001) and DNase I (Sigma-Aldrich 11284932001) with sterile plunger of 3 mL syringe to pass dissociated tissue through 100-micron cell strainer (Corning 352360). Cells were stained with live/dead stain (Thermo Fisher L34966), Fc block (BioLegend 101320) and surface stained with antibodies for XCR1, CD11b, CD11c, Ly6G, MHC-II, CD3, CD19 (BioLegend 148220, 101261, 117318, 127627, 107606, 100233, and 115545, respectively) (RRID:AB_2566410, AB_2572121, AB_493568, AB_10897944, AB_313321, AB_2561387, and AB_2562136, respectively) and Ly6C and CD64 (BD 755198 and 569507, respectively) (RRID:AB_3099650 and AB_3685116, respectively) in Brilliant stain buffer (BD 566349). Cells were fixed and permeabilized with Foxp3 staining kit (Invitrogen 00-5523-00) before intracellular staining for IL-12 p35 (Invitrogen MA5-23559, RRID:AB_2609031) and IL-23 p19 (BD 565317, RRID:AB_2739178). Data was collected using a BD Symphony A3 flow cytometer (BD) and analyzed using FlowJo Software (BD).

For LEGENDplex, whole lungs were homogenized using 70-micron cell strainer (Corning 352350) and plunger of 3 mL syringe into complete RPMI. Single-cell suspensions were pelleted at 1200 rpm for 5 minutes at 4°C. Supernatants were collected for LEGENDplex with pre-made mouse inflammation panel (13-plex) (BioLegend 740446). Prior to final wash and running on flow cytometer, beads were fixed with 4% PFA and plate transferred to BSL1. Data was collected using a BD Symphony A3 flow cytometer (BD) and analyzed using LEGENDplex Data Analysis Software Suite (BioLegend).

### Lung homogenate protein concentration measurements

Whole lungs were homogenized in complete RPMI-1640 media supplemented with 10% FBS, 1 mM sodium pyruvate (Sigma-Aldrich S8636), 0.01 mM HEPES, 1% GlutaMAX (Thermo Scientific 35050061), 1% non-essential amino acids (Gibco 11140-050), and 55 μM 2-mercaptoethanol (Gibco 21985-023) and meshed through 70-micron cell strainer (Corning 352350) with plunger of 5 mL syringe. Cells were pelleted at 1500 rcf for 5 minutes, and supernatants were collected for measuring protein concentrations with BioLegend LEGENDplex mouse inflammation panel (13-plex) (BioLegend 740446).

### Exogeneous IL-17A administration

Mice were intranasally administered 1 μg endotoxin-free recombinant murine IL-17A (R&D Systems, 7956-ML-100/CF) in 40 μL sterile PBS daily or 40 μL of vehicle control daily, or intraperitoneally administered 2 μg endotoxin-free recombinant murine IL-17A in 100 μL sterile PBS daily or 100 uL of vehicle control daily from days 11–20 post aerosol *M. tuberculosis* challenge.

MSA-IL-17A was generated by fusing a (GGGGS)3 linker between the C-terminus of mature murine serum albumin and N-terminus of mature murine IL-17A. MSA-IL-17A was purchased from GenScript USA, Inc in endotoxin-free pH 7.2 PBS. Mice were intranasally administered 5.54 μg MSA-IL-17A (equimolar to 1 μg recombinant murine IL-17A) in 40 μL sterile PBS daily or 40 μL vehicle control, or intraperitoneally administered 11.09 μg MSA-IL-17A (equimolar to 2 μg recombinant murine IL-17A) in 100 μL PBS or 100 μL vehicle control daily between days 11–20 post aerosol *M. tuberculosis* challenge.

### Bone marrow-derived macrophage infection

Male or female WT C57BL/6J BMDMs were incubated on tissue-culture plates for 48 hours prior to infection at 37°C, 5% CO_2_. BMDMs were spin-fected at 1200 rpm for 10 minutes at room temperature for an MOI (multiplicity of infection) of 10. BMDMs were washed with 1× PBS (pH 7.4) and incubated with fresh BMDM media at 37°C, 5% CO_2_ for 24 hours. 24 hours post infection, cultured supernatants were extracted for IL-23 ELISA (BioLegend 433704).

### Statistical analysis

All data were analyzed using FlowJo v10 (FlowJo, LLC) and/or Prism 10 (GraphPad Software). All graphs were analyzed using the Mann–Whitney *U* test. Significant values are symbolized with asterisks of *<0.05, **<0.01, and ns for nonsignificant.

## Data Availability

All data generated or analyzed during this study that are not included in the manuscript and supporting files is available on Dryad (https://doi.org/10.5061/dryad.70rxwdcfm). The following dataset was generated: ZilinskasA BalakhmetA FoxD NiM AgudeloC SamaniH StanleySA 2026*Mycobacterium tuberculosis* suppresses protective Th17 responses during infectionDryad Digital Repository10.5061/dryad.70rxwdcfm

## Additional files

MDAR checklist

## References

[bib1] Aguilo N, Alvarez-Arguedas S, Uranga S, Marinova D, Monzón M, Badiola J, Martin C (2016). Pulmonary but not subcutaneous delivery of BCG vaccine confers protection to tuberculosis-susceptible mice by an interleukin 17-dependent mechanism. The Journal of Infectious Diseases.

[bib2] Andersen P, Andersen AB, Sørensen AL, Nagai S (1995). Recall of long-lived immunity to *Mycobacterium tuberculosis* infection in mice. Journal of Immunology.

[bib3] Arbues A, Aguilo JI, Gonzalo-Asensio J, Marinova D, Uranga S, Puentes E, Fernandez C, Parra A, Cardona PJ, Vilaplana C, Ausina V, Williams A, Clark S, Malaga W, Guilhot C, Gicquel B, Martin C (2013). Construction, characterization and preclinical evaluation of MTBVAC, the first live-attenuated *M. tuberculosis*-based vaccine to enter clinical trials. Vaccine.

[bib4] Augenstreich J, Arbues A, Simeone R, Haanappel E, Wegener A, Sayes F, Le Chevalier F, Chalut C, Malaga W, Guilhot C, Brosch R, Astarie-Dequeker C (2017). ESX-1 and phthiocerol dimycocerosates of *Mycobacterium tuberculosis* act in concert to cause phagosomal rupture and host cell apoptosis. Cellular Microbiology.

[bib5] Barczak AK, Avraham R, Singh S, Luo SS, Zhang WR, Bray M-A, Hinman AE, Thompson M, Nietupski RM, Golas A, Montgomery P, Fitzgerald M, Smith RS, White DW, Tischler AD, Carpenter AE, Hung DT (2017). Systematic, multiparametric analysis of *Mycobacterium tuberculosis* intracellular infection offers insight into coordinated virulence. PLOS Pathogens.

[bib6] Bekmurzayeva A, Sypabekova M, Kanayeva D (2013). Tuberculosis diagnosis using immunodominant, secreted antigens of *Mycobacterium tuberculosis*. Tuberculosis.

[bib7] Casanova JL, Abel L (2002). Genetic dissection of immunity to mycobacteria: the human model. Annual Review of Immunology.

[bib8] Comas I, Chakravartti J, Small PM, Galagan J, Niemann S, Kremer K, Ernst JD, Gagneux S (2010). Human T cell epitopes of *Mycobacterium tuberculosis* are evolutionarily hyperconserved. Nature Genetics.

[bib9] Cox JS, Chen B, McNeil M, Jacobs WR (1999). Complex lipid determines tissue-specific replication of *Mycobacterium tuberculosis* in mice. Nature.

[bib10] Crosskey TD, Beckham KSH, Wilmanns M (2020). The ATPases of the mycobacterial type VII secretion system: Structural and mechanistic insights into secretion. Progress in Biophysics and Molecular Biology.

[bib11] Dijkman K, Aguilo N, Boot C, Hofman SO, Sombroek CC, Vervenne RAW, Kocken CHM, Marinova D, Thole J, Rodríguez E, Vierboom MPM, Haanstra KG, Puentes E, Martin C, Verreck FAW (2021). Pulmonary MTBVAC vaccination induces immune signatures previously correlated with prevention of tuberculosis infection. Cell Reports. Medicine.

[bib12] Dileepan T, Linehan JL, Moon JJ, Pepper M, Jenkins MK, Cleary PP (2011). Robust antigen specific th17 T cell response to group A Streptococcus is dependent on IL-6 and intranasal route of infection. PLOS Pathogens.

[bib13] Dye C, Glaziou P, Floyd K, Raviglione M (2013). Prospects for tuberculosis elimination. Annual Review of Public Health.

[bib14] Enriquez AB, Sia JK, Dkhar HK, Goh SL, Quezada M, Stallings KL, Rengarajan J (2022). *Mycobacterium tuberculosis* impedes CD40-dependent notch signaling to restrict Th_17_ polarization during infection. iScience.

[bib15] Fine PE (1995). Variation in protection by BCG: implications of and for heterologous immunity. Lancet.

[bib16] Flynn JL, Chan J, Triebold KJ, Dalton DK, Stewart TA, Bloom BR (1993). An essential role for interferon gamma in resistance to *Mycobacterium tuberculosis* infection. The Journal of Experimental Medicine.

[bib17] Gaffen SL (2009). Structure and signalling in the IL-17 receptor family. Nature Reviews. Immunology.

[bib18] Gaffen SL, Jain R, Garg AV, Cua DJ (2014). The IL-23-IL-17 immune axis: from mechanisms to therapeutic testing. Nature Reviews. Immunology.

[bib19] Gideon HP, Hughes TK, Tzouanas CN, Wadsworth MH, Tu AA, Gierahn TM, Peters JM, Hopkins FF, Wei JR, Kummerlowe C, Grant NL, Nargan K, Phuah JY, Borish HJ, Maiello P, White AG, Winchell CG, Nyquist SK, Ganchua SKC, Myers A, Patel KV, Ameel CL, Cochran CT, Ibrahim S, Tomko JA, Frye LJ, Rosenberg JM, Shih A, Chao M, Klein E, Scanga CA, Ordovas-Montanes J, Berger B, Mattila JT, Madansein R, Love JC, Lin PL, Leslie A, Behar SM, Bryson B, Flynn JL, Fortune SM, Shalek AK (2022). Multimodal profiling of lung granulomas in macaques reveals cellular correlates of tuberculosis control. Immunity.

[bib20] Gopal R, Monin L, Slight S, Uche U, Blanchard E, Fallert Junecko BA, Ramos-Payan R, Stallings CL, Reinhart TA, Kolls JK, Kaushal D, Nagarajan U, Rangel-Moreno J, Khader SA (2014). Unexpected role for IL-17 in protective immunity against hypervirulent *Mycobacterium tuberculosis* HN878 infection. PLOS Pathogens.

[bib21] Guglani L, Khader SA (2010). Th17 cytokines in mucosal immunity and inflammation. Current Opinion in HIV and AIDS.

[bib22] Guo B, Chang EY, Cheng G (2008). The type I IFN induction pathway constrains Th17-mediated autoimmune inflammation in mice. The Journal of Clinical Investigation.

[bib23] Hinman AE, Jani C, Pringle SC, Zhang WR, Jain N, Martinot AJ, Barczak AK (2021). *Mycobacterium tuberculosis* canonical virulence factors interfere with a late component of the TLR2 response. eLife.

[bib24] Hsu T, Hingley-Wilson SM, Chen B, Chen M, Dai AZ, Morin PM, Marks CB, Padiyar J, Goulding C, Gingery M, Eisenberg D, Russell RG, Derrick SC, Collins FM, Morris SL, King CH, Jacobs WR (2003). The primary mechanism of attenuation of bacillus Calmette-Guerin is a loss of secreted lytic function required for invasion of lung interstitial tissue. PNAS.

[bib25] Ji DX, Witt KC, Kotov DI, Margolis SR, Louie A, Chevée V, Chen KJ, Gaidt MM, Dhaliwal HS, Lee AY, Nishimura SL, Zamboni DS, Kramnik I, Portnoy DA, Darwin KH, Vance RE (2021). Role of the transcriptional regulator SP140 in resistance to bacterial infections via repression of type I interferons. eLife.

[bib26] Jong RM, Van Dis E, Berry SB, Nguyenla X, Baltodano A, Pastenkos G, Xu C, Fox D, Yosef N, McWhirter SM, Stanley SA (2022). Mucosal vaccination with cyclic dinucleotide adjuvants induces effective T cell homing and IL-17-dependent protection against *Mycobacterium tuberculosis* infection. Journal of Immunology.

[bib27] Karbalaei Zadeh Babaki M, Soleimanpour S, Rezaee SA (2017). Antigen 85 complex as a powerful *Mycobacterium tuberculosis* immunogene: Biology, immune-pathogenicity, applications in diagnosis, and vaccine design. Microbial Pathogenesis.

[bib28] Khader SA, Pearl JE, Sakamoto K, Gilmartin L, Bell GK, Jelley-Gibbs DM, Ghilardi N, deSauvage F, Cooper AM (2005). IL-23 compensates for the absence of IL-12p70 and is essential for the IL-17 response during tuberculosis but is dispensable for protection and antigen-specific IFN-gamma responses if IL-12p70 is available. Journal of Immunology.

[bib29] Khader SA, Bell GK, Pearl JE, Fountain JJ, Rangel-Moreno J, Cilley GE, Shen F, Eaton SM, Gaffen SL, Swain SL, Locksley RM, Haynes L, Randall TD, Cooper AM (2007). IL-23 and IL-17 in the establishment of protective pulmonary CD4+ T cell responses after vaccination and during *Mycobacterium tuberculosis* challenge. Nature Immunology.

[bib30] Krueger JG, Eyerich K, Kuchroo VK, Ritchlin CT, Abreu MT, Elloso MM, Fourie A, Fakharzadeh S, Sherlock JP, Yang Y-W, Cua DJ, McInnes IB (2024). IL-23 past, present, and future: a roadmap to advancing IL-23 science and therapy. Frontiers in Immunology.

[bib31] Kumar P (2022). Corrigendum: a perspective on the success and failure of BCG. Frontiers in Immunology.

[bib32] Lazarevic V, Chen X, Shim J-H, Hwang E-S, Jang E, Bolm AN, Oukka M, Kuchroo VK, Glimcher LH (2011). T-bet represses T(H)17 differentiation by preventing Runx1-mediated activation of the gene encoding RORγt. Nature Immunology.

[bib33] Madan-Lala R, Peixoto KV, Re F, Rengarajan J (2011). *Mycobacterium tuberculosis* Hip1 dampens macrophage proinflammatory responses by limiting toll-like receptor 2 activation. Infection and Immunity.

[bib34] Mahairas GG, Sabo PJ, Hickey MJ, Singh DC, Stover CK (1996). Molecular analysis of genetic differences between *Mycobacterium bovis* BCG and virulent *M. bovis*. Journal of Bacteriology.

[bib35] McNab F, Mayer-Barber K, Sher A, Wack A, O’Garra A (2015). Type I interferons in infectious disease. Nature Reviews. Immunology.

[bib36] Nathan A, Beynor JI, Baglaenko Y, Suliman S, Ishigaki K, Asgari S, Huang C-C, Luo Y, Zhang Z, Lopez K, Lindestam Arlehamn CS, Ernst JD, Jimenez J, Calderón RI, Lecca L, Van Rhijn I, Moody DB, Murray MB, Raychaudhuri S (2021). Multimodally profiling memory T cells from a tuberculosis cohort identifies cell state associations with demographics, environment and disease. Nature Immunology.

[bib37] Ogongo P, Tran A, Marzan F, Gingrich D, Krone M, Aweeka F, Lindestam Arlehamn CS, Martin JN, Deeks SG, Hunt PW, Ernst JD (2024). High-parameter phenotypic characterization reveals a subset of human Th17 cells that preferentially produce IL-17 against *M. tuberculosis* antigen. Frontiers in Immunology.

[bib38] Ogongo P, Wassie L, Tran A, Columbus D, Huffaker J, Sharling L, Ouma G, Ouma SG, Bobosha K, Lindestam Arlehamn CS, Gandhi NR, Auld SC, Rengarajan J, Day CL, Queiroz A, Araújo-Pereira M, Fukutani E, Andrade BB, Altman JD, Blumberg HM, Ernst JD (2026). Rare variable *M. tuberculosis* antigens induce predominant Th17 responses in human infection. JCI Insight.

[bib39] Pepper M, Linehan JL, Pagán AJ, Zell T, Dileepan T, Cleary PP, Jenkins MK (2010). Different routes of bacterial infection induce long-lived TH1 memory cells and short-lived TH17 cells. Nature Immunology.

[bib40] Ramgolam VS, Sha Y, Jin J, Zhang X, Markovic-Plese S (2009). IFN-beta inhibits human Th17 cell differentiation. The Journal of Immunology.

[bib41] Sakai S, Kauffman KD, Sallin MA, Sharpe AH, Young HA, Ganusov VV, Barber DL (2016). CD4 T cell-derived IFN-γ plays a minimal role in control of pulmonary *Mycobacterium tuberculosis* infection and must be actively repressed by PD-1 to prevent lethal disease. PLOS Pathogens.

[bib42] Sallin MA, Sakai S, Kauffman KD, Young HA, Zhu J, Barber DL (2017). Th1 differentiation drives the accumulation of intravascular, non-protective CD4 T cells during tuberculosis. Cell Reports.

[bib43] Schlitzer A, McGovern N, Teo P, Zelante T, Atarashi K, Low D, Ho AWS, See P, Shin A, Wasan PS, Hoeffel G, Malleret B, Heiseke A, Chew S, Jardine L, Purvis HA, Hilkens CMU, Tam J, Poidinger M, Stanley ER, Krug AB, Renia L, Sivasankar B, Ng LG, Collin M, Ricciardi-Castagnoli P, Honda K, Haniffa M, Ginhoux F (2013). IRF4 transcription factor-dependent CD11b+ dendritic cells in human and mouse control mucosal IL-17 cytokine responses. Immunity.

[bib44] Sia JK, Bizzell E, Madan-Lala R, Rengarajan J (2017). Engaging the CD40-CD40L pathway augments T-helper cell responses and improves control of *Mycobacterium tuberculosis* infection. PLOS Pathogens.

[bib45] Sleep D, Cameron J, Evans LR (2013). Albumin as a versatile platform for drug half-life extension. Biochimica et Biophysica Acta.

[bib46] Solans L, Aguiló N, Samper S, Pawlik A, Frigui W, Martín C, Brosch R, Gonzalo-Asensio J (2014). A specific polymorphism in *Mycobacterium tuberculosis* H37Rv causes differential ESAT-6 expression and identifies WhiB6 as a novel ESX-1 component. Infection and Immunity.

[bib47] Soldevilla P, Vilaplana C, Cardona PJ (2022). Mouse models for *Mycobacterium tuberculosis* pathogenesis: show and do not tell. Pathogens.

[bib48] Stadhouders R, Lubberts E, Hendriks RW (2018). A cellular and molecular view of T helper 17 cell plasticity in autoimmunity. Journal of Autoimmunity.

[bib49] Stanley SA, Raghavan S, Hwang WW, Cox JS (2003). Acute infection and macrophage subversion by *Mycobacterium tuberculosis* require a specialized secretion system. PNAS.

[bib50] Stanley SA, Johndrow JE, Manzanillo P, Cox JS (2007). The Type I IFN response to infection with *Mycobacterium tuberculosis* requires ESX-1-mediated secretion and contributes to pathogenesis. Journal of Immunology.

[bib51] Stritesky GL, Yeh N, Kaplan MH (2008). IL-23 promotes maintenance but not commitment to the Th17 lineage. Journal of Immunology.

[bib52] Sullivan BM, Jobe O, Lazarevic V, Vasquez K, Bronson R, Glimcher LH, Kramnik I (2005). Increased susceptibility of mice lacking T-bet to infection with *Mycobacterium tuberculosis* correlates with increased IL-10 and decreased IFN-gamma production. Journal of Immunology.

[bib53] Sun M, Phan JM, Kieswetter NS, Huang H, Yu KKQ, Smith MT, Liu YE, Wang C, Gupta S, Obermoser G, Maecker HT, Krishnan A, Suresh S, Gupta N, Rieck M, Acs P, Ghanizada M, Chiou S-H, Khatri P, Boom WH, Hawn TR, Stein CM, Mayanja-Kizza H, Davis MM, Seshadri C (2024). Specific CD4^+^ T cell phenotypes associate with bacterial control in people who “resist” infection with *Mycobacterium tuberculosis*. Nature Immunology.

[bib54] Tameris MD, Hatherill M, Landry BS, Scriba TJ, Snowden MA, Lockhart S, Shea JE, McClain JB, Hussey GD, Hanekom WA, Mahomed H, McShane H (2013). Safety and efficacy of MVA85A, a new tuberculosis vaccine, in infants previously vaccinated with BCG: a randomised, placebo-controlled phase 2b trial. Lancet.

[bib55] Watson RO, Manzanillo PS, Cox JS, Extracellular M (2012). Extracellular *M. tuberculosis* DNA targets bacteria for autophagy by activating the host DNA-sensing pathway. Cell.

[bib56] Watson RO, Bell SL, MacDuff DA, Kimmey JM, Diner EJ, Olivas J, Vance RE, Stallings CL, Virgin HW, Cox JS (2015). The cytosolic sensor cGAS detects *Mycobacterium tuberculosis* DNA to induce type I interferons and activate autophagy. Cell Host & Microbe.

[bib57] Wells RM, Jones CM, Xi Z, Speer A, Danilchanka O, Doornbos KS, Sun P, Wu F, Tian C, Niederweis M (2013). Discovery of a siderophore export system essential for virulence of *Mycobacterium tuberculosis*. PLOS Pathogens.

[bib58] (2022). Global Tuberculosis Report 2022. https://www.who.int/publications/i/item/9789240061729.

[bib59] Yeh WI, McWilliams IL, Harrington LE (2014). IFNγ inhibits Th17 differentiation and function via Tbet-dependent and Tbet-independent mechanisms. Journal of Neuroimmunology.

[bib60] Zhang B, Xiao L, Qiu Q, Miao L, Yan S, Zhou S (2021). Association between IL-18, IFN-γ and TB susceptibility: a systematic review and meta-analysis. Annals of Palliative Medicine.

[bib61] Zhou F, Zhang D (2023). Recent advance in the development of tuberculosis vaccines in clinical trials and virus-like particle-based vaccine candidates. Frontiers in Immunology.

