# Supplementary material for: Mycobacterium tuberculosis suppresses protective Th17 responses during infection
Source: eLife. 2026 Oct 23;15:RP110008. doi: 10.7554/eLife.110008 (PMC13694095; doi:10.7554/eLife.110008)
Supplement: Supplementary file 3 [file elife-110008-fig3-figsupp1-data2.zip › Figure 3-figure supplement 1-source data 2/Figure 3-figure supplement 1-source data 2.pdf]

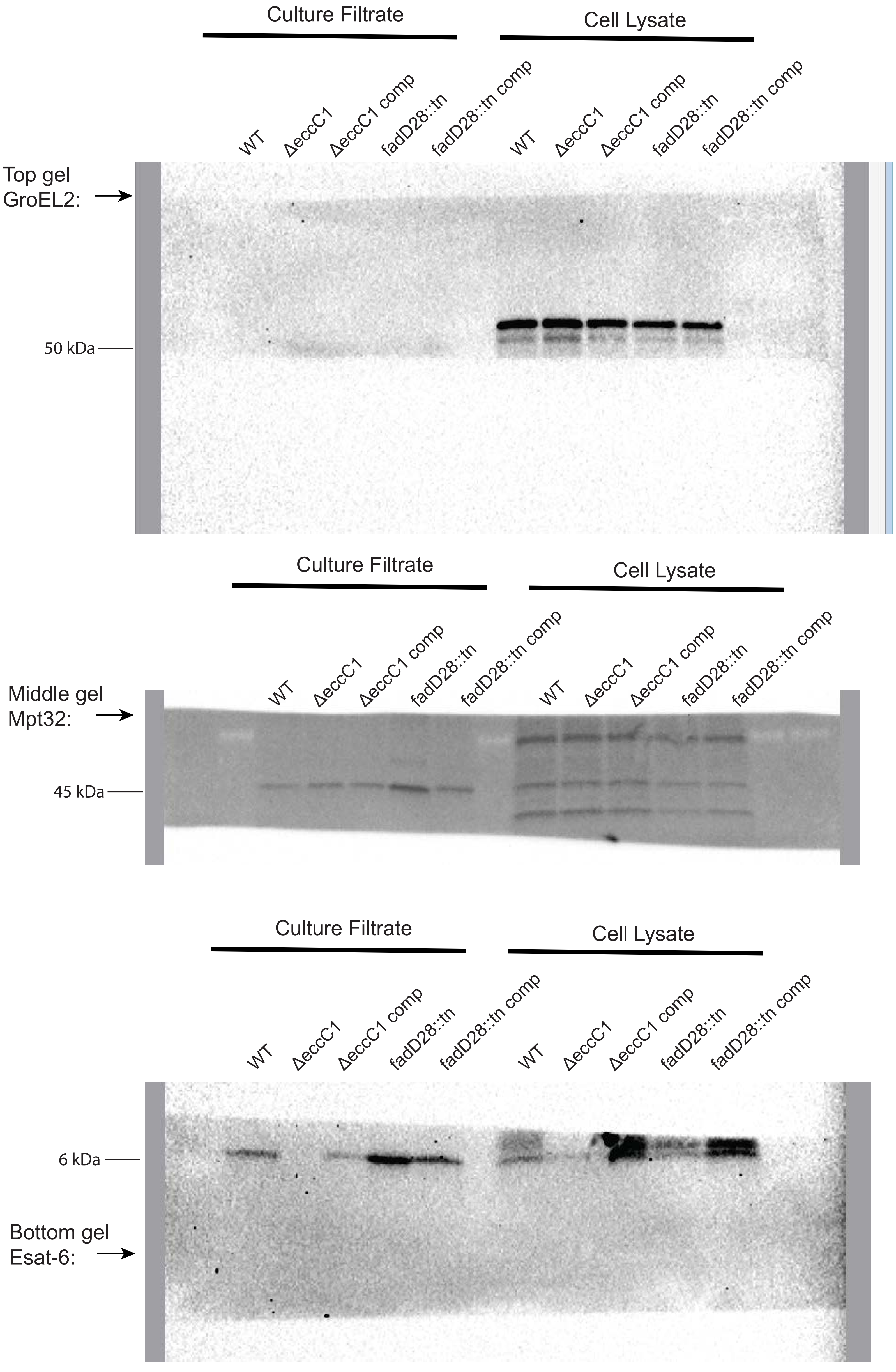
Culture Filtrate
Cell Lysate
WT
ΔeccC1
ΔeccC1 comp
fadD28::tn
fadD28::tn comp
WT
ΔeccC1
ΔeccC1 comp
fadD28::tn
fadD28::tn comp
Top gel
GroEL2:
50 kDa
Culture Filtrate
Cell Lysate
WT
ΔeccC1
ΔeccC1 comp
fadD28::tn
fadD28::tn comp
WT
ΔeccC1
ΔeccC1 comp
fadD28::tn
fadD28::tn comp
Middle gel
Mpt32:
45 kDa
Culture Filtrate
Cell Lysate
WT
ΔeccC1
ΔeccC1 comp
fadD28::tn
fadD28::tn comp
WT
ΔeccC1
ΔeccC1 comp
fadD28::tn
fadD28::tn comp
6 kDa
Bottom gel
Esat-6:
