# Supplementary material for: Mycobacterium tuberculosis suppresses protective Th17 responses during infection
Source: eLife. 2026 Oct 23;15:RP110008. doi: 10.7554/eLife.110008 (PMC13694095; doi:10.7554/eLife.110008)
Supplement: Peer review history

# Editor report: eLife Assessment

**DOI:** 10.7554/eLife.110008.3.sa0

**Authors:** Bavesh D Kana

**Affiliations:** University of the Witwatersrand South Africa

**Keywords:** Important, Solid

This **important** study investigates the mechanisms by which *Mycobacterium tuberculosis* suppresses protective Th17 differentiation during infection. Using ESX-1- and PDIM-deficient mutants of *M. tuberculosis*, which lack functional *eccC1* and *fadD28*, respectively, the authors demonstrate that these virulence factors actively restrict IL-17 responses via a T-bet-dependent pathway, independent of overall bacterial attenuation or infection duration. While the precise molecular interactions by which ESX-1 and PDIM modulate Th17 differentiation warrant further elucidation, the experiments are rigorously designed, the findings are clearly presented, and the evidence is **solid** for a specific role for these factors in limiting protective IL-17 immunity in *M. tuberculosis* infection.

# Referee report: Reviewer #1 (Public review)

**DOI:** 10.7554/eLife.110008.3.sa1

Summary:

The manuscript examines the factors that restrict the induction of IL-17-producing T cells during *Mycobacterium tuberculosis* (Mtb) infection. The authors show that neither infectious route, nor duration of infection are responsible. But they do show that mice that lack the Th1-defining transcription factor, a finding consistent with prior reports in the field of immunology. They also show that 2 highly attenuated Mtb mutants in ESX-1 and PDIM, two well-known Mtb virulence factors, do induce IL-17 producing T cells. In contrast, Mtb mutants in mmpl4 are also similarly attenuated, but do not induce IL-17-producing T cells, suggesting that this property is not simply a result of attenuation but due to specific properties of ESX-1 and PDIM-deficient mutants.

Strengths:

(1) It is interesting that mice infected with ESX-1 and PDIM mutants have increased induction of Th17 cells.

(2) Data is solid and convincing throughout.

Weaknesses:

There are two main criticisms:

(1) B6 mice, compared to humans are known to be very Th1 skewed and the Th1 transcription factor T-bet is known to be a strong inhibitor of Th17 responses. Thus, these Th17 inhibitory factors may be stronger in B6 mice than humans, as many humans do make Th17 responses to Mtb infection.

(2) The molecular insights about how Th17 induction is somewhat limited. Tbet induction is known to restrict Th17 development and this is a t cell intrinsic mechanism. In contrast, the IL-23 association revealed seems to be extrinsic to T cells and to act on T cells. It is not clear these factors related to each other in restricting Th17 induction.

Additional points:

(1) The manuscript states, "Under the conditions where Th17s are highly induced, mice infected with either ΔESX-1 or PDIM lacking Mtb, the Il17a-/- mice had ~3-5 fold higher CFU than WT mice (Figures 3F-G). These results indicate that the induction of Th17s is not dependent on the attenuation of Mtb in general, but instead Mtb utilizes ESX-1 and PDIM to suppress the induction of a Th17 response that enhances protection against Mtb infection." One consideration, however, is that ESX-1, PDIM, and mmpl4 mutants all have similarly reduced CFUs in the lung, but have different CFUs in the lung-draining LN where T cell priming occurs? The bacterial burden in the LN may be more important for regulating T-bet, IL-23, and Th17 differentiation, since the LN is where T cell priming occurs, than the CFU in the lung. Perhaps ESX-1 and PDIM mutants have reduced CFU in the LN, but mmpl4 does not. This difference in LN burdens may be the primary driver of Th17 priming, as high avidity interactions are thought to be an important driver of T-bet induction. Thus, without examining the LN, some questions remain regarding the conclusion that the altered Th17 response in the attenuated strains is not due to the attenuation itself. However, I agree the CFU in the LN probably reflects that in the lung, and if so, the author's conclusions would be sound.

(2) Do LN cDC1 and high levels of IL-12 p35 manifest in mice infected with the mmpl4 mutant? Likewise do LN cDC2's express low levels of IL-12 p19 (akin to those infected with WT Mtb). If these observations for ESX-1 and PDIM mutants are mechanistically linked to the increased numbers of Th17 cells, then you would expect mice infected with mmpl4 mutants to be more like those infected with WT Mtb than to those infected with ESX-1 and PDIM mutants. These experiments would help provide more convincing evidence that the identified mechanisms are due specifically to outcomes regarding Th17 induction. However, I agree the author's conclusions are the most likely explanation given the current data.

# Referee report: Reviewer #2 (Public review)

**DOI:** 10.7554/eLife.110008.3.sa2

In this manuscript, the authors tackle an important question of why IL-17 production and TH17 responses are lower than expected during Mtb infection. The authors identify an axis of cross-regulation between TH1 and TH17 cells and provide data to support roles for Mtb virulence factors ESX1 and PDIM in promoting TH1 responses and/or suppressing TH17 responses.

Strengths:

The strengths include the significance of the work, the combination of host and Mtb genetic models to dissect the mechanistic basis for regulation of IL-17 production from T cells during infection, and the rigor of the experiments. There are a number of exciting findings from the work, including the cross talk between T cell responses and the impact of ESX1 and PDIM on these responses. It is particularly striking that that IL17a deficient mice partially rescue the attenuation of ESX-1 and PDIM mutants.

Comments on revised version.

The revised manuscript has tempered a lot of the language in the original text to more accurately state (and not overstate) interpretations of the data. The claim that the effect is independent of route of infection seems a little too large of a claim when only two routes were tested (aerosol and intranasal). And although the authors revised the results section to acknowledge the contribution of an IFNg-dependent suppression of IL-17 production from T cells, the abstract has not been updated and still claims that all effects are independent of IFNg.

# Referee report: Reviewer #3 (Public review)

**DOI:** 10.7554/eLife.110008.3.sa3

Summary:

The manuscript by Zilinskas et al seeks to understand the mechanisms underlying the ability of Mtb to suppress Th17 differentiation. As Th17 responses are needed for protective immunity against TB, this is an important topic of investigation. They use Mtb mutants that lack *eccC1* (from ESX-1 locus) and *fadD28* (encoding PDIM) and implicate a Tbet-dependent pathway by which Mtb modulates Th17 differentiation. The mechanism by which ESX-1/PDIM function to impact Th17 differentiation is, however, unclear, which limits the novelty of the results.

Strengths:

Understanding how Mtb limits Th17 differentiation has implications for vaccine development. Comparative study of KO mice and Mtb mutants is a strength.

Weaknesses:

(1) Addressing several questions related to the Tbet KO mouse experiments would strengthen the study. Do the Tbet KO mice have elevated IL-4/5/13 (which has been previously reported in non-TB studies) in addition to IL-17? The lack of Th17 cells in the IFNg KO compared to the Tbet KO may reflect a difference in timing, since only 3-week data are shown; earlier and later time points would provide a better interpretation. The authors do not present any data on neutrophil infiltration in WT vs Tbet KO vs IFNg KO mice. Since IL-17 is known to be important for recruiting neutrophils to the lung, neutrophil data are important for clarifying the mechanism underlying the CFU outcomes.

(2) While IL-23 is important for sustaining IL-17 production, IL-6, TGF-b and/or IL-1β are necessary for Th17 polarization. What were the levels of these cytokines in DCs in the lung? (Fig 5). Additionally, Tbet-deficient DCs exhibit impaired activation of antigen-specific Th1 cells and have reduced IL-12 production. Given the data showing higher IL-17 levels in Tbet KO mice, the authors should provide information on the DC phenotype (IL-23, IL-6 etc) in the Tbet KO experiments.

(3) The mechanism by which ESX-1/PDIM function to impact Th17 differentiation is not clear. While data showing a role for ESX-1 and PDIMs in inhibiting Th17 responses is interesting, there is no insight into the potential mechanism of action. Fig 3 showing reduction in IFNg+ CD4 T cells after infection with *eccC1* and *fadD28* mutants suggests that this outcome is due to a lower bacterial load relative to WT Mtb at the 3-week time point. Since IFNg is known to suppress IL-17, the higher levels of Th17 cells could be due to the reduction in IFNg due to the attenuated growth of the mutants.

# Author comment: Author response

**DOI:** 10.7554/eLife.110008.3.sa4

**Authors:** Alex Zilinskas, Amir Balakhmet, Douglas Fox, Heyuan Michael Ni, Carolina Agudelo, Helia Samani, Sarah A Stanley

**Affiliations:** University of California, Berkeley Berkeley United States; University of California, Berkeley Berkeley United States; University of California, Berkeley Berkeley United States; University of California, Berkeley Berkeley United States; University of California, Berkeley Berkeley United States; University of California, Berkeley Berkeley United States; University of California, Berkeley Berkeley United States

The following is the authors’ response to the original reviews.

**Public Reviews:**

**Reviewer #1 (Public review):**

There are two main criticisms:

(1) It is not clear how much the factors uncovered here are true beyond B6 mice. B6 mice, compared to humans, are known to be very Th1-skewed, and Tbet is a strong inhibitor of Th17-specific T cells. Many people make IL-17-producing T cells in response to Mtb infection.

We appreciate the point that not all findings in mice are directly translatable to humans. The B6 mouse is widely used as a model organism for tuberculosis due to its tractability and the wealth of genetic tools available for this strain. While it is true that many individuals do produce Th17 cells after infection with Mtb, humans are still very Th1-dominant, and not all infected individuals produce Th17 cells. We can speculate that the mechanisms outlined in this paper may contribute to the reasons that Th17 responses are not more robust in humans, a finding that may be useful in guiding vaccine design in the future.

(2) Very few novel insights are mechanistically revealed about how Th17 induction is restricted by Mtb. Tbet induction is known to restrict Th17 development, and this is a T-cell intrinsic mechanism. In contrast, the IL-23 association revealed seems to be extrinsic to T cells and to act on T cells. How, if at all, are these factors related to each other in restricting Th17 induction? Also, the conclusion that it is not a result of attenuation is not completely convincing.

While it is established that Th1 differentiation can inhibit Th17 differentiation, we believe that rigorously demonstrating this genetically in the context of Mtb infection remains important. Moreover, it cannot be assumed that IL-17 elicited by dampening the Th1 response can lead to enhanced control of infection. We view addressing this as a significant contribution. Furthermore, we also show that the ESX-1 and PDIM virulence factors are functionally linked by suppression of IL-17 responses. The effect is unlikely to be simply due to attenuation of the strains as an equally attenuated control strain does not elicit Th17 cells. We believe that these insights are both novel and important for understanding immune responses to Mtb.

Other points:

(1) The authors show that mice infected with a deficiency in ESX-1 have more IL-17-producing CD4 T cells in response to stimulation with an ESAT-6 peptide pool (Figure 3B). Because ESAT-6 is encoded by ESX-1, why do mice infected with this Mtb mutant have any ESAT-6-specific T cells? Is it an incomplete knockdown?

The ESX-1 knock-out *M. tuberculosis* Erdman strain is a Δ*EccC1* mutant. This strain can produce Esat-6 but cannot secrete Esat-6 out of the bacterial cell. Thus Esat-6 protein is present and able to be processed for MHC-II presentation. We also use Ag85b peptide pool stimulation and report similar effects as Esat-6 peptide pool stimulation.

(2) The manuscript states, "Under the conditions where Th17s are highly induced, mice infected with either ΔESX-1 or PDIM lacking Mtb, the Il17a-/- mice had ~3-5 fold higher CFU than WT mice (Figures 3F-G). These results indicate that the induction of Th17s is not dependent on the attenuation of Mtb in general, but instead Mtb utilizes ESX-1 and PDIM to suppress the induction of a Th17 response that enhances protection against Mtb infection." I don't think the last sentence is necessarily true. I can imagine a scenario in which the induction of the Th17s is, in fact, due to the attenuation, and the Th17 induction still contributes to protection.

We tested another attenuated *M. tuberculosis* strain with no known relationship with ESX-1 or PDIM, ΔMmpL4. This attenuated mutant fails to induce IL-17A–producing CD4 T cells to the same extent as observed in mice infected with ESX-1-deficient or PDIM-deficient strains, which is strong evidence that simple attenuation of virulence does not result in higher numbers of Th17 cells being elicited.

(3) ESX-1, PDIM, and mmpl4 mutants all have similarly reduced CFUs in the lung, but what about the LN? The bacterial burden in the LN may be more important for regulating T-bet, IL-23, and Th17 differentiation, since the LN is where T cell priming occurs, than the CFU in the lung. Perhaps ESX-1 and PDIM mutants have reduced CFU in the LN, but mmpl4 does not. This difference in LN burdens may be the primary driver of Th17 priming, as high avidity interactions are thought to be an important driver of T-bet induction.

We acknowledge that this is a formal possibility, however we maintain that the phenotype is specific to ESX and PDIM mutants, rather than MmpL4 mutants. Even if this phenotype arises from a tissue-specific attenuation of ESX/PDIM mutants, it remains a specific phenotype of these mutants, and not all attenuated mutants, albeit less directly. More importantly, the observation that these mutants induce higher levels of the Th17-polarizing cytokine IL-23 from infected cells ex vivo suggests that this is not an indirect phenomenon.

(4) Do LN cDC1 and high levels of IL-12 p35 in mice infected with the mmpl4 mutant? Likewise, LN cDC2's express low levels of IL-12 p19 (akin to those infected with WT Mtb)? If these observations for ESX-1 and PDIM mutants are mechanistically linked to the increased numbers of Th17 cells, then you would expect mice infected with mmpl4 mutants to be more like those infected with WT Mtb than those infected with ESX-1 and PDIM mutants.

Because ΔMmpL4 and complemented strains resulted in T cell profiles that were not different from the wild-type, we did not measure mediastinal lymph node dendritic cell expression of IL-12 p35 and IL-23 p19 in infections with these mutants.

(5) ESX-1 and PDIM are very different virulence factors - a protein secretory pathway and cell wall lipid, respectively? Mechanistically, how would mutants in these pathways give very similar outcomes regarding Th17 cells unless it was simply as an aspect of their attenuation? Perhaps, mmpl4 mutants simply differ in some aspects of their attenuation, such as bacterial burdens in LNs, or their interaction with cDCs?

We are not the first to link phenotypes of ESX-1 and PDIM. Both systems have both been shown to be important for *M. tuberculosis* permeabilization of the host cell phagosome after phagocytosis, and for suppression of type I IFN responses, among other responses. Thus, these seemingly different virulence factors clearly work together to support specific virulence traits during infection. The exact mechanism of how ESX-1 and PDIM interact is not completely understood and is an area for future investigation.

**Reviewer #2 (Public review):**

The following conclusions and interpretations should be revisited, rephrased, and re-evaluated:

(1) The manuscript neglects to analyze T cell responses in the dLN, which is the critical site where these responses are initiated (only DC cytokine production is measured in the dLN). The differences in the lungs could reflect trafficking of T cells to the lungs, local lung T cell responses, or durability of the T cell responses in the lungs. The authors state in the last results section that "These results indicate that the ESX-1 and PDIM virulence factors impact naïve T cell differentiation at the draining mediastinal lymph node..." but T cell responses are never measured in the dLN.

Due to the limited size of the mediastinal lymph node at 3 weeks post infection, we were unable to obtain enough cells for both myeloid cell analysis and T cell analysis, as we perform staining for these panels separately due to the decrease in viability of myeloid cells observed during T cell restimulation. In addition, because T cells in the lung are the population of cells most critical for mediating the outcome of infection, we believe analyzing the T cell response in the lymph nodes though interesting, is not crucial for this study. We have edited the manuscript to be clearer, as suggested by the reviewer.

(2) Figure 2: The authors state that "Importantly, IFN-γ deficient mice did not exhibit elevated levels of IL-17A producing CD4 T cells demonstrating that IFN-γ production is not the mechanism by which Th1 T cells limit a Th17 response during Mtb infection", but the difference is significantly different and even more obvious in Panel B. In fact, if the Panel D y-axis was on a log scale, the Ifng-/- would likely look more like Tbet-/- than WT. Based on this data, it seems like IFNg is having an effect and should not be completely discounted. Does the deletion of Ifng affect the number of Tbet+ T cells?

We agree that the IFN-γ^-/-^ have only 5x more IL-17 producing CD4 T cells than WT mice while Tbet^-/-^mice exhibit a 25-fold increase compared to WT. We have added this information to the text, and now point out that IFN-γ production is not the sole mechanism by which Th1 T cells limit a Th17 response during Mtb infection.

In addition, the deletion of Tbet results in an increased number of IFNg+IL-17+ double positive T cells (Figure 2B), in addition to a sizable IFNg single positive T cell population maintained in the Tbet-/- mice (10x the negative control of Ifng-/-). Is this why Tbet deletion is not as severe as Ifng deletion, because T cells are still making IFNg?

It is possible that the residual IFN-γ produced by T-bet-deficient animals contributes to their relatively modest susceptibility to infection. However, our data show that deletion of IL-17 in this background renders T-bet–deficient mice nearly as susceptible as IFN-γ deficient mice, arguing that the remaining IFN-γ is not a major protective factor.

Along these lines, the statement in the text that, "Tbet-/-Il17a-/- mice completely lacked both IFN-γ producing...." T cells is not supported by the data in Figure 2C. Tbet-/-Il17a-/- mice look to have more gamma-producing T cells than Tbet-/- mice (which is already 10x the negative control of Ifng-/- in panel 2B if one includes the gamma single positive and IFNg/IL-17 double positive).

We have amended the language in the text to be more consistent with the data.

(3) In the Results sections describing Figures 3, 4, and 5, the authors equate IL-17 production by T cells with TH17 responses and IFNg expression with TH1, but Tbet and RORgt expression in the T cells should be measured to make conclusions about TH1 and TH17. Or the authors can rephrase their findings to specifically state the observations as IFNg or IL-17 expressing CD4+ T cells.

We believe that calling a CD4 T cell in the lung that is producing IFN-γ (and not IL-17) a Th1 cell is appropriate. Potentially confounding cells include those which also produce IL17, which we have ruled out, or T_FH_ cells that may be common in lymph nodes but are not common in lungs at this time point and under these conditions.

(4) Conceptually, do the authors think that ESX1/PDIM promotes TH1 responses and this blocks TH17 or are ESX1/PDIM blocking TH17 responses directly, allowing for increased TH1 responses? It would be helpful to clarify the model in this regard, describe how the data supports one model or the other, and then make sure the language is consistent throughout. Can these effects on T cell responses be tested and recapitulated in vitro using infected APC and T cell co-cultures?

While it is possible that PDIM and ESAT-6 suppress Th17 through promotion of Th1 differentiation, we do not have data to support this model currently. However, we have added a comment making this point to the discussion.

**Reviewer #3 (Public review):**

Weaknesses:

(1) The authors should acknowledge and reference key findings from the literature that have identified suppression of Th17 differentiation as an Mtb virulence mechanism, e.g., the role of the Hip1 protease and CD40 signaling (Madan-Lala JI 2014, Sia Plos Path 2017, Enriquez iScience 2022) and Khader JI 2005, showing the requirement of IL-23 for Th17 responses in vivo in a TB mouse model.

We thank the reviewer for pointing these references out and have added them to the discussion section of the manuscript.

(2) Addressing several questions related to the Tbet KO mouse experiments would strengthen the study. Do the Tbet KO mice have elevated IL-4/5/13 (which has been previously reported in non-TB studies) in addition to IL-17? The lack of Th17 cells in the IFNg KO compared to the Tbet KO may be due to a difference in timing, since only 3-week data are shown; earlier and later time points would provide better interpretation. The authors do not present any data on neutrophil infiltration in WT vs Tbet KO vs IFNg KO mice. Since IL-17 is known to be important for recruiting neutrophils to the lung, data on neutrophils are important for clarifying the mechanism for the CFU outcomes.

We agree that it is surprising that, in the context of TB, Th17 responses are protective whereas excessive neutrophil recruitment is detrimental to the host. In IFN-γ–deficient mice, neutrophils are recruited and contribute to the increased susceptibility of this strain (PMID: 21967766). In separate work from our lab, we have shown that the phenotype of neutrophils recruited to the lungs during Mtb infection influences disease outcome (PMID: 40937719). It is possible that differences in the host environment and the timing of the response shape the effects of neutrophils on the host; these and the other questions raised by the reviewer will be the subject of future studies.

(3) While IL-23 is important for sustaining IL-17 production, IL-6, TGF-b and/or IL-1β are necessary for Th17 polarization. What were the levels of these cytokines in DCs in the lung? (Figure 5). Additionally, Tbet-deficient DCs exhibit impaired activation of antigen-specific Th1 cells and have reduced IL-12 production. Given the data showing higher IL-17 levels in Tbet KO mice, the authors should provide information on the DC phenotype (IL-23, IL-6, etc.) in the Tbet KO experiments.

While these are interesting points, investigating mechanisms of Tbet-dependent suppression of IL-17 is beyond the scope of this study.

(4) The mechanism by which ESX-1/PDIM function to impact Th17 differentiation is not clear. While data showing a role for ESX-1 and PDIMs in inhibiting Th17 responses is interesting, there is no insight into the potential mechanism of action. Figure 3 showing reduction in IFNg+ CD4 T cells after infection with *eccC1* and *fadD28* mutants suggests that this outcome is due to a lower bacterial load relative to WT Mtb at the 3-week time point. Since IFNg is known to suppress IL-17, the higher levels of Th17 cells could be due to the reduction in IFNg due to the attenuated growth of the mutants. Additionally, what was the level of Type I IFNs elicited by these mutants?

We included the MmpL4 knockout Mtb Erdman strain as a control to ensure that attenuation of mutants is not the cause of the increase in IL-17. We also showed that eliminating type I IFN signaling by deleting its receptor has minimal impact on Th17 differentiation, even in the context of a host that produces excess type I IFN. Therefore we do not believe that type I IFN elicited by these mutants is explanatory for the phenotype.

(5) Since macrophages have been implicated in the reduced cytokines seen in the ESX-1 mutant, IL-23 and other cytokine data on lung macrophages would complement the DC data.

Because dendritic cells are primarily responsible for priming CD4 T cell responses, we believe that this result in macrophages would not substantially alter our conclusions. That said, it was demonstrated previously that macrophages infected with ESX-1 mutants produce less IL-12p40, a subunit of IL-23.

(6) Figure 5. There are many fewer DCs overall in the *eccC1* and *fadD28* mutant groups, which could account for the increased % IL-23p19 in DCs (5D). What were the levels of IL-23 in DC1s?

The amount of IL-23 p19+ in type I conventional dendritic cells (cDC1s) was near zero as shown in supplementary figure 6A. cDC1s are known to not express IL-23 p19 in mice.

**Recommendations for the authors:**

**Reviewer #2 (Recommendations for the authors):**

(1) What do the authors mean by the alternative secretion part of "ESX1 Type VII alternative secretion system" that they refer to?

Bacterial alternative secretion systems facilitate the export of proteins from the bacterial cell independent of the canonical Sec-dependent secretion system required for export of most secreted bacterial proteins across the inner membrane. However to avoid confusion, we have removed the word alternative.

(2) Not sure naïve fits in this sentence at the end of the introduction: "Furthermore, we observe a strong Th17 response during infection with ΔESX-1 or PDIM lacking Mtb in naïve mice....".

We have removed the word Naïve.

(3) Figure legend for 1A-C says analysis performed at 21 dpi, but the figure shows the time course.

We have corrected this error.

**Reviewer #3 (Recommendations for the authors):**

(1) Figure 1 should show the non-stimulated flow plot.

We have added the unstimulated samples.

(2) The % IL-17 in the flow plots is not consistent across Figures 1, 2, and 3. Not sure why the scales for the Y-axis for IL-17 differ so much between Figures 1 and 2/3. IS there a technical issue with compensation?

We did not experience any difficulties with compensations. These experiments were done over several years of work. For every experiment, new single-color controls were used and gating was done with the FMO gating strategy. Minor variation such as we see here is not surprising.

(3) Discuss Yeh et al J Neuroimmunol 2014- show that IFNγ inhibits Th17 differentiation and function via Tbet-dependent and Tbet-independent mechanisms.

We have added this reference to the manuscript.
